# Classification of industrial chemicals for respiratory chemosensory irritation using the TRPV1-expressing neuronal SH-SY5Y cell model and machine learning

**DOI:** 10.1007/s00204-025-04288-6

**Published:** 2026-01-22

**Authors:** María Hinojosa, Gunnar Johanson, Ulf Norinder, Anna Forsby

**Affiliations:** 1 Department of Biochemistry and Biophysics, Stockholm University, Stockholm, Sweden; 2 Institute of Environmental Medicine, Karolinska Institute, Stockholm, Sweden; 3 MTM Research Centre, School of Science and Technology, Örebro University, Örebro, Sweden; 4 Department of Pharmaceutical Biosciences, Uppsala University, Uppsala, Sweden

**Keywords:** Respiratory sensory irritation, RD_50_, SH-SY5Y, TRPV1, Calcium influx, Random forest

## Abstract

Respiratory sensory irritation is the basis for many occupational exposure limits. Irritation thresholds have hitherto mainly been identified in humans by questionnaires and in mice by measuring the inhaled concentration causing 50% respiratory depression (RD50). Both methods are ethically questionable. We investigated an alternative New Approach Methodology (NAM) approach, namely the neuronal SH-SY5Y cell model expressing the sensory receptor TRPV1 in combination with random forest-based machine learning. The intracellular Ca2+ concentration was monitored during acute exposure to different concentrations of 34 organic chemicals. Potency and efficacy were determined with and without the TRPV1 antagonist capsazepine (CZ). Fifteen of the chemicals induced TRPV1 activation at some concentrations, however only phenol appeared as a true TRPV1 agonist. Using machine learning, the parameters EC20, Emax, concentration at Emax, the two first components from principal component analyses, and pH were analysed against previously published RD50 data to classify each chemical as non-irritant/irritant or as non-irritant/ intermediate/irritant. The best 2-class model (accuracy 0.90, outlier frequency 4.8%) was the one using experiments with CZ present, suggesting that the irritancy was not mediated by TRPV1 activation. The best 3-class model (accuracy 0.77, outlier frequency 5.7%) was the one using data without CZ, indicating that TRPV1 activation may play a role for intermediate irritation. The three false negative chemicals, as predicted by the NAMs, were the most irritating chemicals according to RD50 determined in vivo, indicating that other processes may also be important.

**Supplementary Information:**

The online version contains supplementary material available at 10.1007/s00204-025-04288-6.

## Introduction

The human chemosensory system plays an important role for the detection and protection against many airborne environmental pollutants (Lehmann et al. 2016). From a regulatory perspective, respiratory sensory irritation in the eyes and upper airways is regarded as an adverse effect (Brüning et al. 2014). For example, around two thirds of the substances for which an occupational exposure limits (OEL) have been established are sensory irritants (Nielsen and Wolkoff 2017). Further, it has been estimated that 40% of the OELs are set primarily to prevent irritation (Dick and Ahlers 1998; Edling and Lundberg 2000; Gaffney and Paustenbach 2007; Brüning et al. 2014). Typical sensory irritating volatiles include acids, aldehydes, and amines. Many of these chemicals may occur not only in the work environment but also in e.g. consumer products (Gupta et al. 2015; Lehmann et al. 2016).

Sensory irritation by airborne compounds is perceived via stimulation of the trigeminal nerve through the C- and Aδ-fibers that innervate the facial skin, the tongue, the cornea and the mucosae of eyes, mouth, and nose (Lehmann et al. 2016). Several types of receptors and ion channels are involved in the response. Acid sensing ion channels (ASICs), cholinergic receptors and the family of multimodal TRP cation channels play significant roles (Caterina et al. 1997; Thuerauf et al. 1999; Patapoutian et al. 2003; Story et al. 2003; Deval et al. 2010; Lehmann et al. 2016; Hummel and Frasneli 2019).

The vanilloid receptor type 1 (TRPV1) is a non-selective, Ca^2+^ permeable, cation channel, which is expressed primarily in sensory neurons throughout the body (Caterina et al. 1997). Activation by temperatures above 42 °C as well as chemical stimuli lead to Ca^2+^ influx into the cytosol, both from the extracellular space and intracellular stores (Gallego-Sandín et al. 2009). The influx of cations causes activation of the sensory neural pathways resulting in a sensation of hotness or pain (Julius 2013). Furthermore, the Ca^2+^ influx induces an efferent signaling cascade in the peripheral sensory C-fibers by triggering exocytosis of neuropeptides, leading to neurogenic inflammation in the surrounding tissues and sensitization of the nociceptors (Berridge et al. 1998; Gouin et al. 2017). The principal agonist to the TRPV1 ion channel is capsaicin, the pungent component in chili peppers (Caterina and Julius 1999; Hummel and Frasneli 2019), but the receptor can be activated by diverse compounds. For instance, it has been shown that surfactants as well as other irritating chemicals such as formaldehyde also act as agonists to the TRPV1 ion channel (Lilja et al. 2007; Forsby et al. 2012; Han et al. 2012; Lindegren et al. 2009; Tian et al. 2009).

Respiratory irritancy is a well-known effect of volatile chemicals. As air contaminants, they have been studied extensively in humans over the years, using controlled chamber studies as the golden standard (Nielsen and Wolkoff 2017). Due to cost and ethical consideration, chamber studies are typically conducted with few healthy subjects, who are acutely exposed to the test chemical for minutes to hours. The responses are then monitored by recording symptoms via interviews, questionnaires and/or rating scales. Several variables may modify the outcome, including e.g. exposure duration, age, gender, and individual susceptibility (Nielsen and Wolkoff 2017). Furthermore, most human studies only used one or a few exposure levels, making the No-Observed Adverse Effect Level (NOAEL) and the Lowest Observed Adverse Effect Level (LOAEL) estimates uncertain.

The most common animal method to assess respiratory sensory irritation is the so-called Alarie test, which determines the airborne concentration at which the substance causes a 50% reduction in breathing frequency (RD_50_) in mice (Alarie 1966; Nielsen and Wolkoff 2017). Although the RD_50_ values have demonstrated a relatively good correlation with irritancy in humans, this method has been criticized for its substantial variability as well as from ethical aspects for the test animals (Nielsen and Wolkoff 2017).

The aim of the present work was to explore an alternative in vitro test to assess respiratory sensory irritation. The human neuroblastoma SH-SY5Y cell line transfected with the TRPV1 receptor has been successfully used for classification of stinging and non-stinging detergents (Forsby et al. 2012). Thus, we decided to assess Ca^2+^ influx in this cell line after acute exposure to compounds with various respiratory irritation potency. Furthermore, the role of TRPV1-mediated response would be confirmed by the blockage of Ca^2+^ influx by the TRPV1 antagonist capsazepine. The efficacy and potency in vitro*,* without and with capsazepine, together with the pH of the compounds, were compared with in vivo irritancy data, namely RD_50_ values in mice.

## Materials and methods

### Chemicals and reagents

**Fig. 1:**
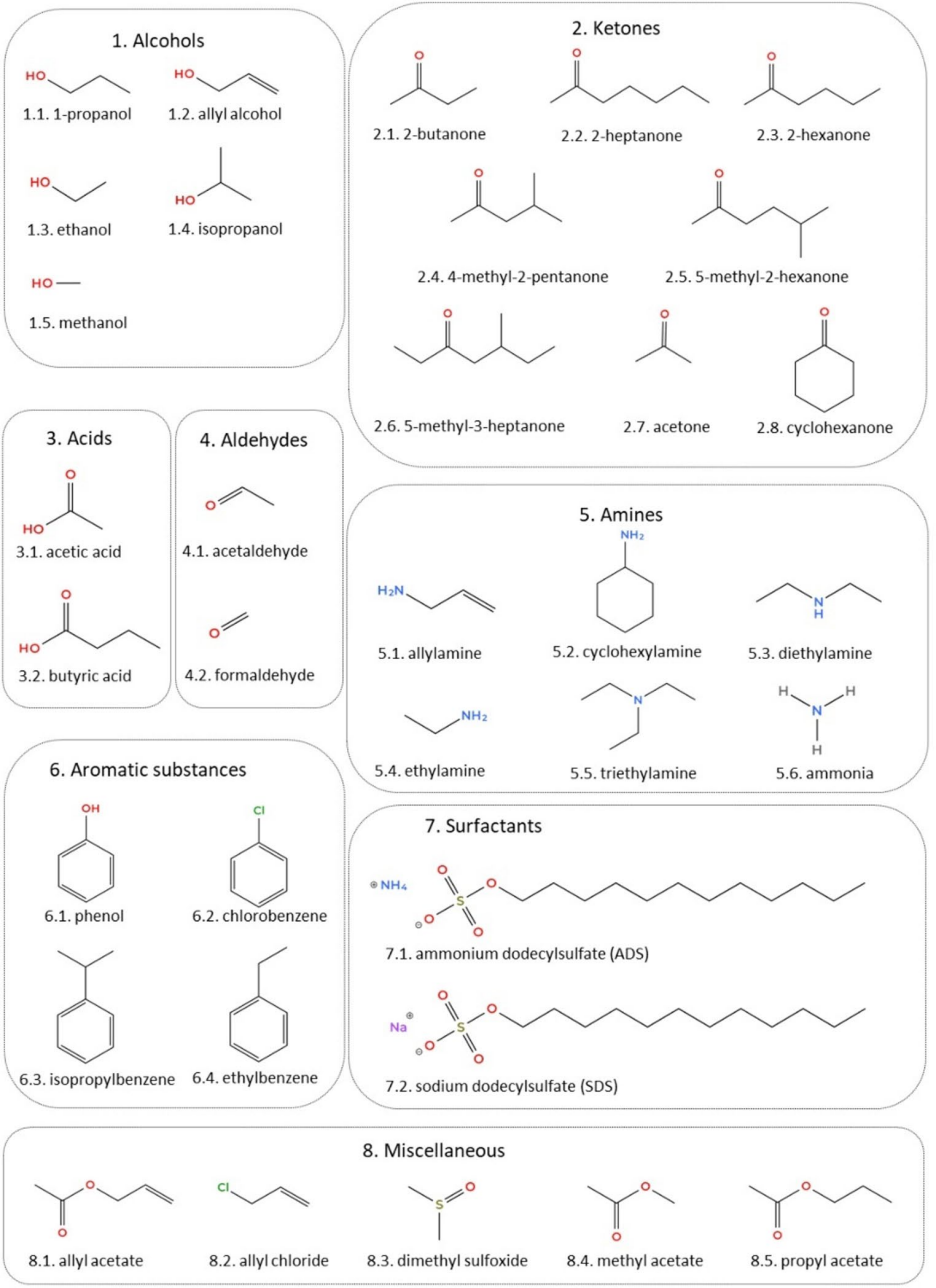
Chemical groups and structures of the compounds studied

All the chemicals, reagents and cell culture material are described in Supplementary Table 1 with their references. The chemical structure of the compounds tested can be found in Fig. 1.

### Cell model and culturing

The human neuroblastoma cell line SH-SY5Y was previously transfected with a rTRPV1-expressing plasmid (Lilja et al. 2007). Cells were stored under liquid nitrogen and used for 10 passages from thawing, and they were routinely screened for mycoplasma infections. The TRPV1-SH-SY5Y cells were cultured in 20 mL Advanced Minimal Essential medium with 2 mM L-glutamine, 100 U/mL penicillin and 100 µg/mL streptomycin, 1% non-essential amino acids, 5% fetal bovine serum and 0.4 µg/mL puromycin, denoted complete AdvMEM. The cells were maintained as monolayer cultures in 75 cm^2^ flasks and incubated in 100% humidity at 37 °C in air with 5% CO_2_. Every seventh day, the cells were subcultivated by aspiring the complete AdvMEM, adding 1 mL TrypLE Express solution for 2 min to detach at 37 °C. Nine mL of complete AdvMEM were added, a single cell suspension was created, and the cell density was determined. A total of 1.5 million cells were added to 20 mL of complete AdvMEM in a 75 cm^2^ flasks and kept at 37 °C in humidified atmosphere with 5% CO_2_. The medium was changed after 3–4 days to 20 mL fresh complete AdvMEM.

### Test solutions

The compounds were aliquoted in 1.5 mL glass vials and tested in 1:3 dilution steps. For some test chemicals additional dilution steps (1:1.5) were inserted to optimize the concentration–response curve fit. All dilutions were prepared at a 6 times higher concentration than the intended final concentration, as a 1:6 dilution took place at the addition of the compounds to the cells in the Flex Station II fluorescence reader. The dilutions were done with modified Krebs–Ringer-Hepes (KRH) buffer [125 mM NaCl, 5 mM KCl, 1.2 mM MgSO_4_ × 7H_2_O, 2 mM CaCl_2_, 1.2 mM KH_2_PO_4_ × 2H_2_O, 5 mM HEPES, and 6 mM glucose, pH 7.4] and vigorous mixing between each dilution step.

### Intracellular calcium measurements

The cells were plated in clear 96-well plates at a density of 3 × 10^4^ cells/well if the test would take place after 4 days, and 4 × 10^4^ cells/well if the experiment took place 3 days after seeding, to yield 90% confluence at the time of measurement. Acute cellular changes in the intracellular free Ca^2+^ concentration, [Ca^2+^]_i_, were measured, using the fluorophore Fura-2 AM, in a semi-high-throughput functional screening fluorescence reader, Flex Station II (Molecular Devices). For this, 20 µL of 12 µM Fura-2 AM diluted in KRH buffer from a stock solution of 2 mM Fura-2 AM dissolved in DMSO, were added to the 100 µL AdvMEM per well to get 2 µM Fura-2 AM in the wells. After 30 min of incubation at 37 °C, the medium with Fura-2 AM was discarded and the cells were gently washed with 200 µL KRH buffer. In rows A-D of the plate, the wells were filled with 100 µL KRH buffer, and in rows E–H with 100 µL KRH buffer with 30 µM capsazepine (dissolved in DMSO). After another 20 min at 37 °C, basal fluorescence was measured at excitation wavelengths 340 nm and 380 nm and emission wavelength 510 nm. Just before the experiment, each concentration of the compounds and capsaicin were transferred to a 96-well compound plate, which was placed in the Flex Station II compound plate compartment. The pH was measured in the solutions at the concentrations applied to the cells. After 30 s, 20 µL of each compound or the positive control (capsaicin dissolved in DMSO, final concentration in the wells 100 nM) were added automatically to one column at the time using the integrated 8-channel pipetting device in the Flex Station II, and the fluorescence was measured every 3 s for another 150 s (measuring 3 min in total). The number of replicates (n) refer to individual experiments performed at different occasions. Each individual experiment was performed in 3 technical replicates.

### Analysis of concentration-fluorescence response

The area under the curve (AUC) of fluorescence intensity was determined during the 150 s of measurement using SoftMax (Molecular Devices). The fluorescence intensity before addition of test chemicals or capsaicin was set to 0% and that after addition of 100 nM capsaicin was set to 100%. The concentration–response data were fitted to a single or biphasic sigmoidal curve fit based on the lowest value of the Akaike information criterion (AIC). In all curve fits, the constants were set to top = the highest value obtained (Emax), and bottom = 0. The concentration that generated 20% increase in [Ca^2+^]_i_ in relation to 100 nM capsaicin (EC_20_), the maximum response (Emax) and the concentration (conc) at Emax, were determined from the curve fit equations. The equivalent values were determined from data that were obtained from exposure with chemicals in the presence of the TRPV1 channel antagonist capsazepine, variables indicated by the suffix + CZ. Statistical analyses (curve fits and t-test with two-stage step-up method of Benjamini, Krieger and Yekutieli (Benjamini et al. 2006) were carried out in GraphPad Prism 10.3.1.

### Principal component analysis

All compounds, except for chlorobenzene, ethylbenzene and isopropylbenzene for which neither significant Emax, nor significant EC_20_ were achieved, were included in the principal component analyses (PCAs). The variables Emax and Emax + CZ were used without transformation, whereas EC_20_, EC_20_ + CZ, conc at Emax and conc at Emax + CZ were transformed to the negative logarithm. The pH at Emax was also included. Three PCAs were performed with variables obtained from (1) the tests without capsazepine added (No CZ; i.e. including the Ca^2+^ influx mediated by TRPV1), (2) the tests with capsazepine added (+ CZ; i.e. excluding the Ca^2+^ influx mediated by TRPV1), and (3) both types of tests (all_invitro = No CZ and + CZ). Two principal components (PCs) were derived from each PCA. The PCAs were performed by using GraphPad Prism 10.3.1 with the “Standardize” setting (i.e. scaled data to have a mean of 0 and standard deviation (SD) of 1).

### In vivo reference data

Historical RD_50_ values were collected from literature to evaluate the data that were obtained from the TRPV1-SH-SY5Y cell model. Several in vivo studies have been performed on different species and the RD_50_ values vary. Herein, the mean of RD_50_ values for mouse with exposure time between 5 to 30 min were used as reference values for evaluation (Schaper 1993). The values for the surfactants ADS and SDS were obtained from Ciuchta and Dodd (1978). As these surfactants are solids at room temperature, they were administered as aerosols and the unit used for the RD_50_ was mg/m^3^. However, the RD_50_ of all other test compounds were given in ppm, a preferable unit as it allows comparisons between compounds on an equimolar basis. To obtain the same metric as for the other test compounds, we translated the RD_50_ values for ADS and SDS from mg/m^3^ to ppm assuming 25 °C and using the formula:

$${\mathrm{Concentration}}\left( {{\mathrm{ppm}}} \right) = 24.45 \times {\text{concentration }}\left( {{\mathrm{mg}}/{\mathrm{m}}^{3} } \right)/{\mathrm{molecular}}\;{\mathrm{weight}}$$

To our knowledge, there is no common classification agreed on irritation potency based on RD_50_ values. For the purpose of this investigation and to be able to derive prediction models, we divided the chemicals in two classes with an RD_50_ cutoff at 700 ppm (Table 1, 2-class model). To make the prediction models more informative, we also tried with a second cutoff at 10,000 ppm (Table 1, 3-class model).

**Table 1:** In vivo respiratory irritancy classification based on average RD_50_ values (Shaper 1993)

2-class model			3-class model		
Class 01	Class 2		Class 0	Class 1	Class 2
> 700 ppm	< 700 ppm		> 10,000 ppm	700–10,000 ppm	< 700 ppm

**Table 2:** Efficacy and potency of the test chemicals on Ca^2+^ influx compared to capsaicin in the TRPV1-SH-SY5Y cells without and with capsazepine

Chemicals ranked in order of potency in each group	EC_20_ (M)	Emax (% of Capsaicin)	Conc. at Emax (M)	EC20 + CZ (M)	Emax + CZ (% of Capsaicin)	Conc. at Emax + CZ (M)	pH of Conc. at Emax
Capsaicin	4.9E-10	100	1.0E-07	N.E	20	1.0E-07	
1. Alcohols							
1.1. 1-propanol	5.0E-04	95	1.5E-01	8.6E-03	66	1.5E-01	6.97
1.2. allyl alcohol	6.8E-02	63	3.2E-01	1.6E-01	47	3.2E-01	6.58
1.3. ethanol	2.2E-01	62	1.7E + 00	5.5E-01	44	1.1E + 00	7.15
1.4. isopropanol	1.4E-01	53	1.3E + 00	2.6E-01	44	1.3E + 00	7.10
1.5. methanol	5.6E-01	59	2.5E + 00	1.3E + 00	46	2.5E + 00	6.95
2. Ketones							
2.1. 2-butanone	4.1E-02	84	1.1E + 00	1.6E-01	58	1.1E + 00	7.32
2.2. 2-heptanone	1.2E-03	66	2.7E-02	1.4E-02	51	2.4E-01	7.29
2.3. 2-hexanone	9.6E-05	59	3.0E-02	3.0E-02	20	3.0E-02	7.01
2.4. 4-methyl-2-pentanone	1.5E-02	84	2.7E-01	7.8E-02	64	2.7E-01	7.39
2.5. 5-methyl-2-hexanone	1.2E-03	66	2.4E-01	3.0E-02	59	2.4E-01	7.40
2.6. 5-methyl-3-heptanone	1.9E-03	59	2.1E-01	3.2E-02	47	2.1E-01	7.29
2.7. acetone	6.5E-03	61	1.5E-01	2.7E-02	34	1.5E-01	7.09
2.8. cyclohexanone	4.1E-03	112	3.2E-01	7.6E-03	81	1.1E-01	7.13
3. Acids							
3.1. acetic acid	6.5E-04	65	2.4E-03	6.8E-04	56	7.2E-03	4.29
3.2. butyric acid	6.7E-08	95	2.1E-06	8.6E-08	75	6.2E-06	7.05
4. Aldehydes							
4.1. acetaldehyde	1.4E-01	110	5.9E-01	2.0E-01	98	5.9E-01	5.89
4.2. formaldehyde	2.4E-03	92	4.0E-01	6.0E-03	57	4.0E-01	11.93
5. Amines							
5.1. allylamine	6.6E-04	134	1.5E-01	9.4E-04	116	4.9E-02	11.50
5.2. cyclohexylamine	2.0E-07	132	1.5E-05	4.0E-07	106	4.4E-05	7.11
5.3. diethylamine	4.4E-07	125	1.6E-05	5.4E-07	95	1.8E-06	7.10
5.4. ethylamine	1.1E-04	109	2.2E-02	2.0E-04	109	2.2E-02	9.26
5.5. triethylamine	2.3E-04	117	7.2E-01	2.9E-04	116	2.4E-01	11.51
5.6. ammonia	1.0E-03	118	2.8E-01	2.0E-03	88	9.3E-02	11.52
6. Aromatic substances							
6.1. phenol	2.7E-05	114	1.3E-02	1.1E-03	100	1.3E-02	6.92
6.2. chlorobenzene	N.E	N.E	N.E	N.E	N.E	N.E	
6.3. isopropylbenzene	N.E	N.E	N.E	N.E	N.E	N.E	
6.4. ethylbenzene	N.E	N.E	N.E	N.E	N.E	N.E	
7. Surfactants							
7.1. ammonium dodecyl sulfate	4.2E-08	133	1.8E-05	5.0E-08	107	1.5E-03	6.88
7.2. sodium dodecyl sulfate	6.9E-05	123	4.3E-03	2.2E-04	120	1.3E-02	7.39
8. Miscellaneous							
8.1. allyl acetate	1.1E-02	52	2.1E-01	8.1E-02	43	2.1E-01	6.97
8.2. allyl chloride	7.6E-03	38	1.5E-02	2.1E-01	32	1.2E + 00	7.25
8.3. dimethyl sulfoxide	3.0E-01	92	1.4E + 00	3.3E-01	63	1.4E + 00	7.11
8.4. methyl acetate	4.5E-03	108	1.3E + 00	2.0E-02	94	2.1E + 00	6.79
8.5. propyl acetate	7.2E-02	65	2.9E-01	1.2E-01	62	2.9E-01	7.43

N.E., no effect

**Table 3:** Accuracy of predicted classification from the random forest analyses and outliers from the KNN analyses of chemicals as irritating or non-irritating (2-class model) and as irritating, moderately irritating or non-irritating (3-class model)

Descriptor sets^§^	Classes*	Explanation^¤^	Accuracy	%outliers in 100 models (5-KNN)	#outliers in 100 models (5-KNN)
all_invitro	2 classes (01 vs 2)	pEC20(M); p(conc at Emax(M)): pH at conc at Emax; Emax (% of capsaicin); pEC20 + CZ(M); p(conc at Emax + CZ(M)); Emax + CZ (% of capsaicin); PC1 scores no CZ; PC2 scores no CZ; PC1 scores + CZ; PC2 scores + CZ; PC1 scores_all_invitro; PC2 scores_all_invitro	0.87	7.1	113
No CZ	2 classes (01 vs 2)	Including TRPV1 activity: pEC20(M)); p(conc at Emax(M)); pH at conc at Emax; Emax (% of capsaicin); PC1 scores no CZ; PC2 scores no CZ	0.81	5.7	91
** +CZ**	**2 classes (01 vs 2)**	**Blocking TRPV1 activity with CZ: pEC20 + CZ(M); p(conc at Emax + CZ(M)); pH at conc at Emax; Emax + CZ (% of capsaicin);PC1 scores + CZ; PC2 scores + CZ)**	**0.90**	**4.8**	**77**
all_invitro	3 classes (0 vs_1_vs__2)	pEC20(M); p(conc at Emax(M)): pH at conc at Emax; Emax (% of capsaicin); pEC20 + CZ(M); p(conc at Emax + CZ(M)); Emax + CZ (% of capsaicin); PC1 scores no CZ; PC2 scores no CZ; PC1 scores + CZ; PC2 scores + CZ; PC1 scores_all_invitro; PC2 scores_all_invitro	0.71	7.1	113
**No CZ**	**3 classes (0 vs_1_vs__2)**	**Including TRPV1 activity: pEC20(M)); p(conc at Emax(M)); pH at conc at Emax; Emax (% of capsaicin); PC1 scores no CZ; PC2 scores no CZ**	**0.77**	**5.7**	**91**
+CZ	3 classes (0 vs_1_vs__2)	Blocking TRPV1 activity with CZ: pEC20 + CZ(M); p(conc at Emax + CZ(M)); pH at conc at Emax; Emax + CZ (% of capsaicin);PC1 scores + CZ; PC2 scores + CZ)	0.71	4.8	77

^**§**^all_invitro; including all descriptors. No CZ; including TRPV1 activity. + CZ; blocking TRPV1 activity with capsazepine

^*^2-class model: class 2; RD50 < 700 ppm, class 01; RD50 > 700 ppm. 3-class model: class 2; RD50 < 700 ppm, class 1; RD50 > 700 ppm and < 10,000 ppm, class 0; RD50 > 10,000 ppm

^¤^Data for each descriptor are presented in Supplementary Table 3

Bold text; best 2- and 3- classification models

They (bold) are the ones with higher accuracy

### Machine learning (random forest) analysis

As for the PCAs, three sets of descriptors were used in the machine learning analyses, i.e. all_invitro, +CZ (only capsazepine-related descriptors, i.e. blockage of TRPV1) and No CZ (no capsazepine-related descriptors, i.e. including TRPV1 activated Ca^2+^ influx), respectively. The analyses also included the scores from PC1 and PC2 from each PCA as descriptors (Supplementary Table S2).

The 3-class dataset was class-wise stratified randomly divided into 100 pairs of training (50%) and test (50%) sets using StratifiedShuffleSplit (scikit-learn v. 0.23.2) (Pedregosa et al. 2011). The same training and test sets as for the 3-class dataset, were used for the binary (2-class) investigations where the non- and intermediate irritants were merged into one class and modeled versus the irritants in the latter case. The compounds of the respective 100 pairs were used for all three descriptor sets. For each of the training sets a Random Forest classification (RandomForestClassifier, scikit-learn v. 0.23.2) model was built using default values for the classifier. The derived models were subsequently used to predict the corresponding test set. For each descriptor set and each compound, the count of predicted classes in all 100 test sets, where the compound was present and inside the applicability domain (see below) of the model, was recorded. The majority class count (expressed as fractions in Tables 3 and 4) from these, at most, 100 predictions, was used as the predicted outcome for each compound.

**Table 4:**
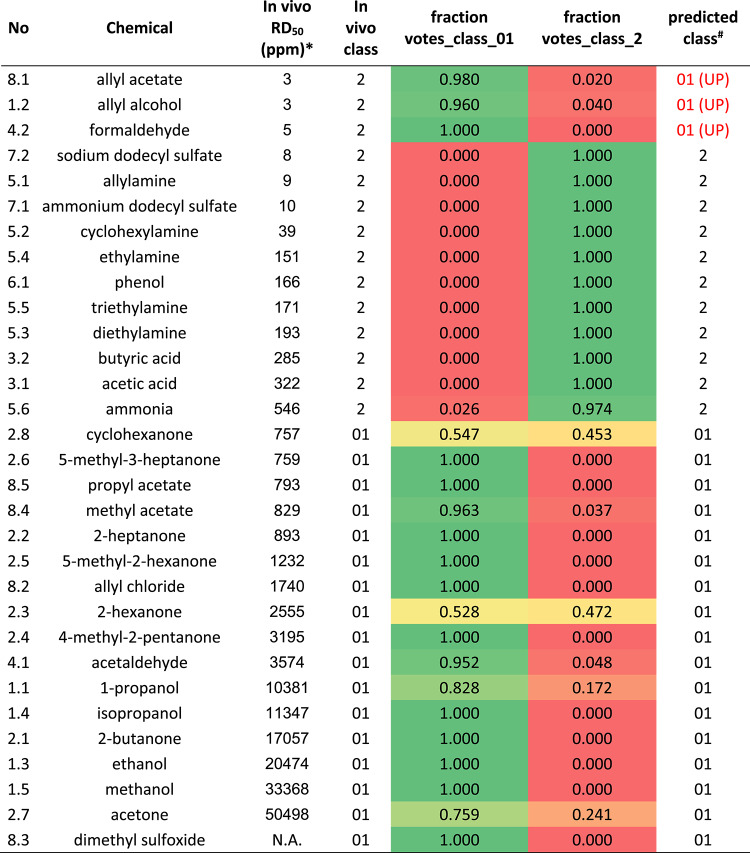
Probability for classification into the 2-class model by using in vitro variables for the best model (+CZ) in the random forest prediction. Heat map indicates fraction of votes in each class (colour figure online)

^*^Average RD_50_ of values reported by Schaper (1993)

^#^UP; under predicted

N.A., not available

Colours indicate the fraction of votes, being in green equal o similar to 1, and in red equal or similar to 0

### Model applicability domain

The applicability domain of each training set derived model was constructed in the following manner: The training set descriptors were scaled to the range 0–1 using the MinMaxScaler (scikit-learn v. 0.23.2). The distances of the 5 nearest neighbours (5-KNN) were determined for each training set compound using NearestNeighbors (scikit-learn v. 0.23.2) and the corresponding mean distance was computed. Finally, the overall mean distance and corresponding SD from all training set compounds mean values were calculated. The cut-off distance for a new test compound to be within the applicability domain of the model was set as the overall mean value plus 2 times the SD, roughly approximating a 95% confidence interval. The applicability domain was tested for each of the derived models and the count of outlier predictions was recorded.

## Results

### Concentration-fluorescence response

**Fig. 2:**
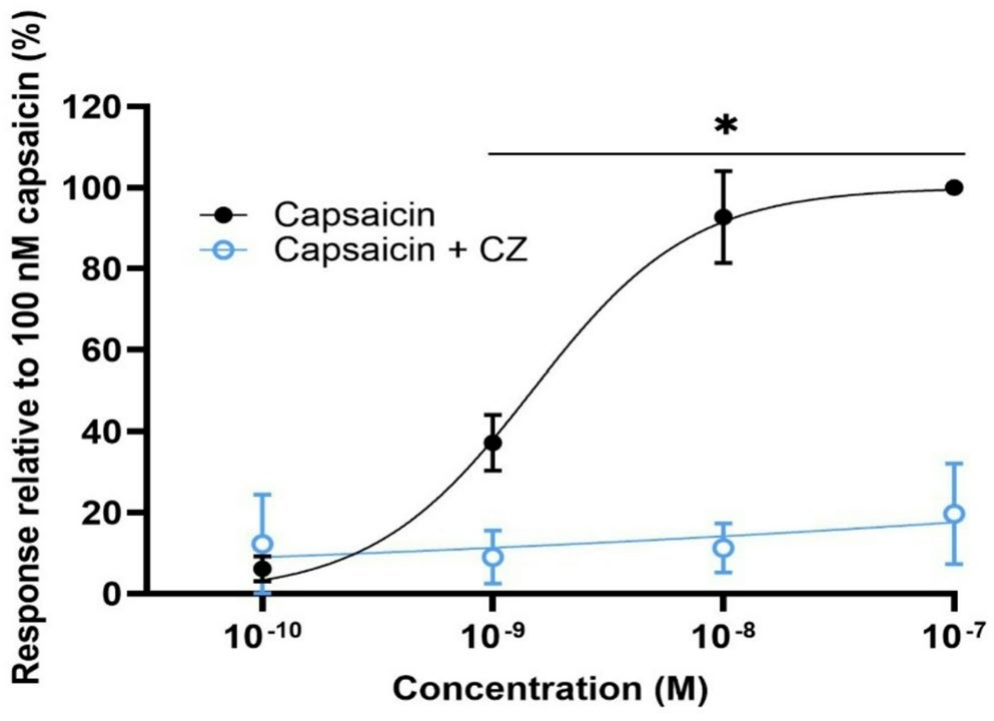
Concentration–response curve of calcium levels after exposure to capsaicin. In black, response of the cells to capsaicin in relation to 100 nM capsaicin. In blue, response of the cells to capsaicin after pre-exposure to 30 µM capsazepine (CZ), in relation to 100 nM capsaicin. Data are presented as mean ± SD. *Significantly different from capsaicin + CZ (*p* < 0.05)

The response to capsaicin was measured as increase in Fura-2 fluorescence due to Ca^2+^ influx in TRPV1 transfected SH-SY5Y cells (Supplementary Fig. 1). The fluorescence response, expressed as AUC, was normalized to the response of 100 nM capsaicin and visualized in a sigmoidal concentration-fluorescence response curve. The Ca^2+^ influx was abolished when capsaicin was applied in the presence of the TRPV1 channel antagonist capsazepine (Fig. 2 and Supplementary Fig. 1).

**Fig. 3:**
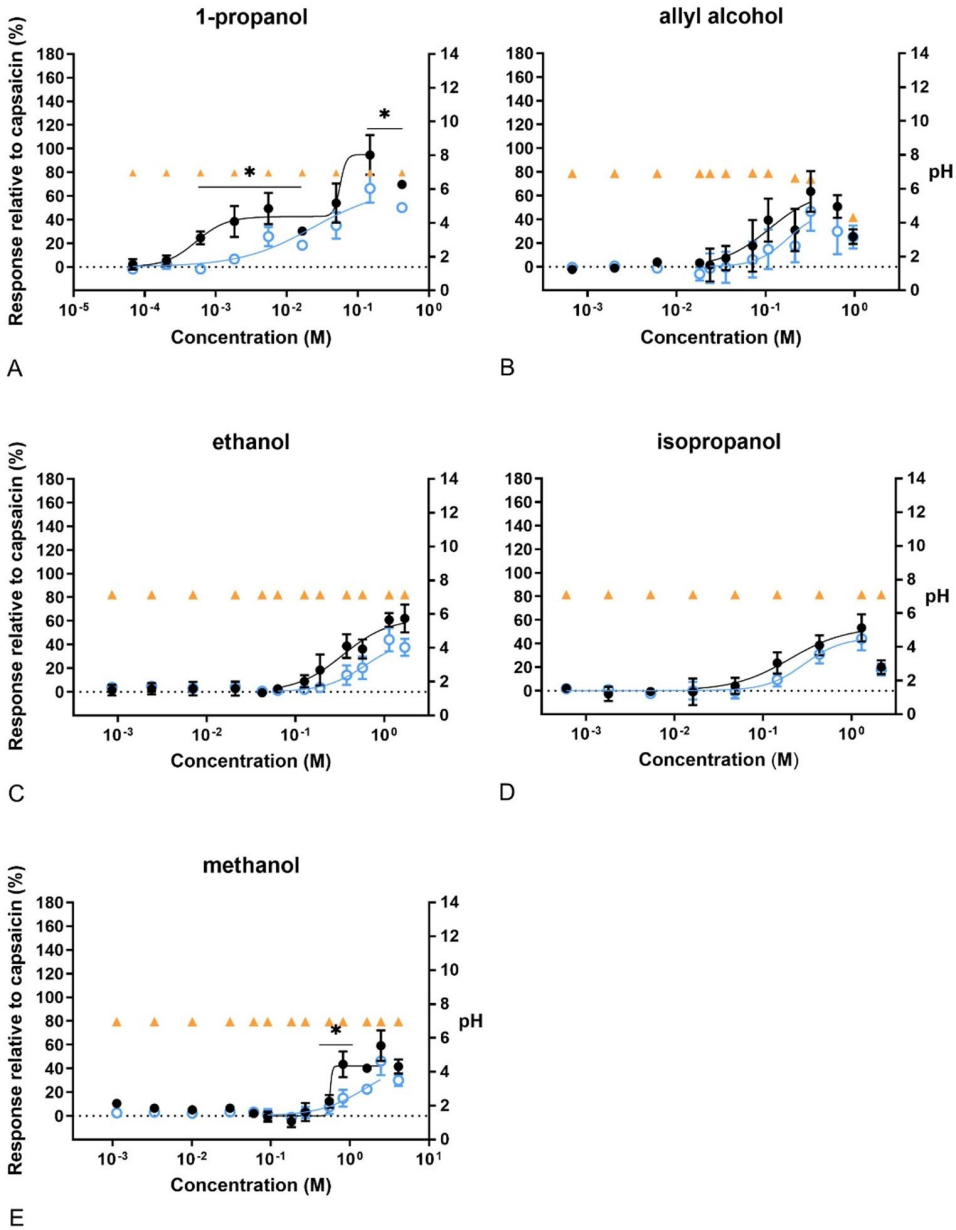
Concentration–response curves of calcium levels measured after exposure to alcohols. In black, response of the cells to the compounds in relation to 100 nM capsaicin. In blue, response of the cells to the compounds after pre-exposure to capsazepine (CZ), related to 100 nM capsaicin. In orange, pH of the final test medium (i.e. the buffer for calcium measurements) at the different concentrations tested. A) 1-propanol, B) allyl alcohol, C) etanol, D) isopropanol, E) methanol. Data are presented as mean ± SD. *significantly different from compund + CZ (*p* < 0.05)

**Fig. 4:**
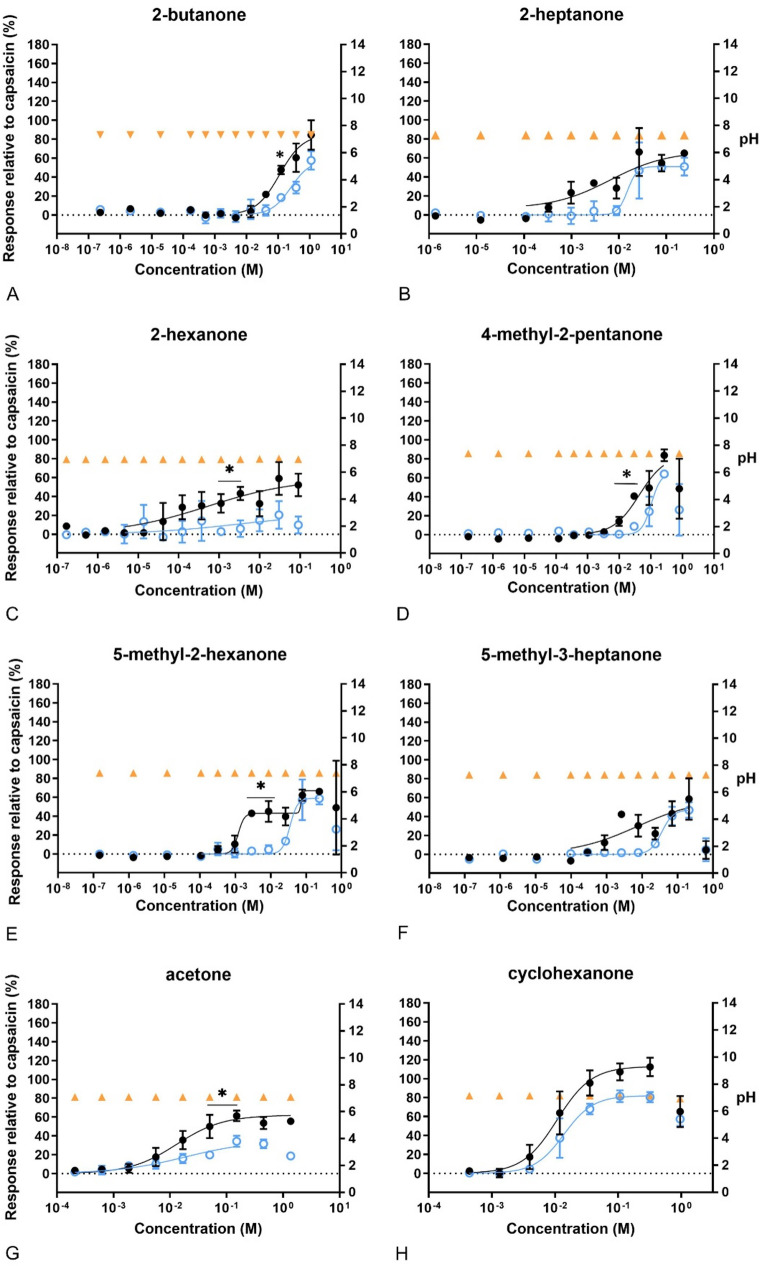
Concentration–response curves of calcium levels measured after exposure to ketones. In black, response of the cells to the compounds compared to 100 nM capsaicin. In blue, response of the cells to the compounds after pre-exposure to capsazepine (CZ), related to 100 nM capsaicin. In orange, pH of the final test medium (i.e. the buffer for calcium measurements) at the different concentrations tested. **A** 2-butanone, **B** 2-heptanone, **C** 2-hexanone, **D** 4-methyl-2-pentanone, **E** 5-methyl-2-hexanone, **F** 5-methyl-3-heptanone, **G** acetone, **H** cyclohexanone. Data are presented as mean ± SD. *Significantly different from compound +CZ (*p* < 0.05)

**Fig. 5:**
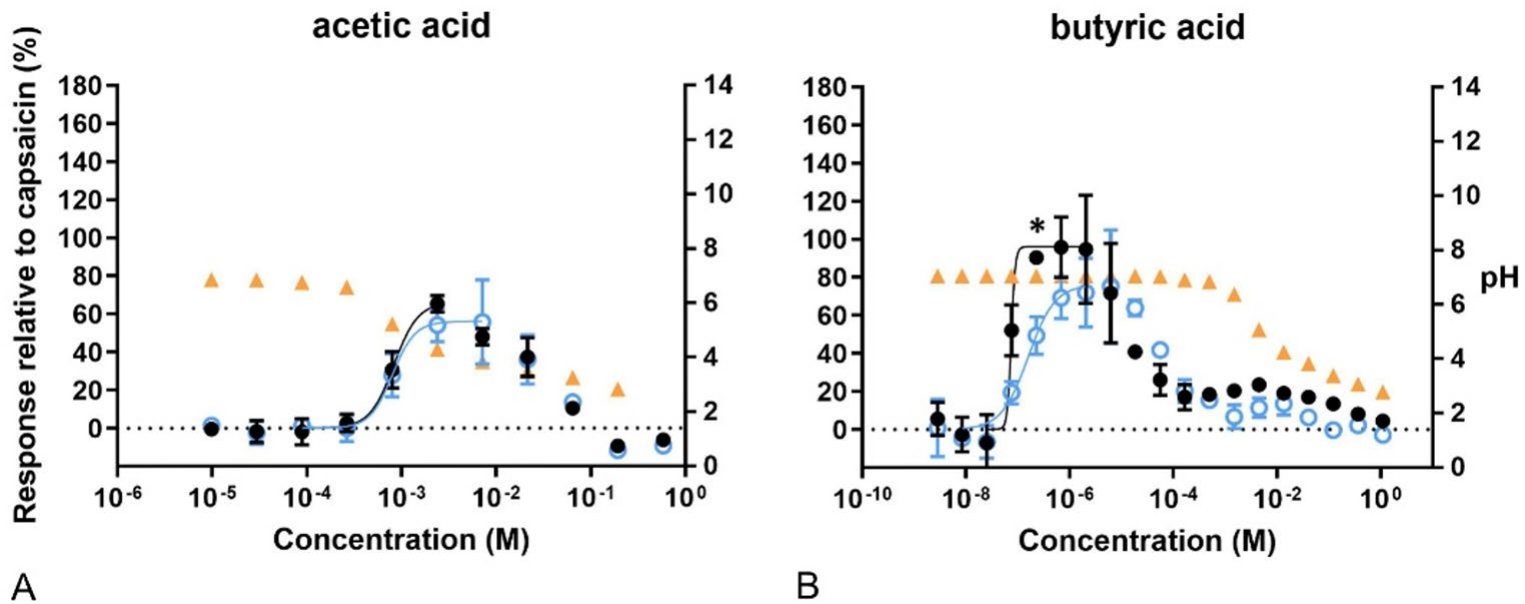
Concentration–response curves of calcium levels measured after exposure to acids. In black, response of the cells to the compounds compared to 100 nM capsaicin. In blue, response of the cells to the compounds after pre-exposure to capsazepine (CZ), related to 100 nM capsaicin. In orange pH of the final test medium (i.e. the buffer for calcium measurements) at the different concentrations tested. A) acetic acid, B) butyric acid. Data are presented as mean ± SD. *Significantly different from compound +CZ (*p* < 0.05)

**Fig. 6:**
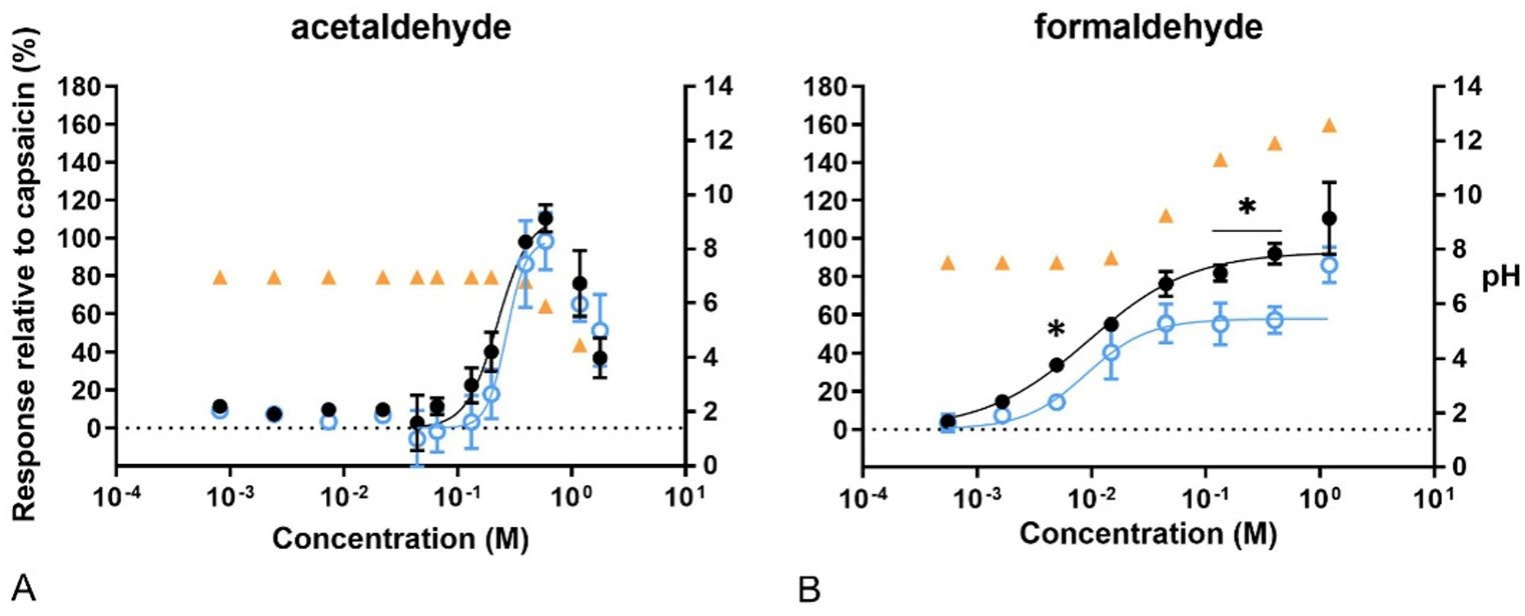
Concentration–response curves of calcium levels measured after exposure to aldehydes. In black, response of the cells to the compounds compared to 100 nM capsaicin. In blue, response of the cells to the compounds after pre-exposure to capsazepine (CZ), related to 100 nM capsaicin. In orange, pH of the final test medium (i.e. the buffer for calcium measurements) at the different concentrations tested. A) acetaldehyde. B) formaldehyde. Data are presented as mean ± SD. *Significantly different from compound +CZ (*p* < 0.05)

**Fig. 7:**
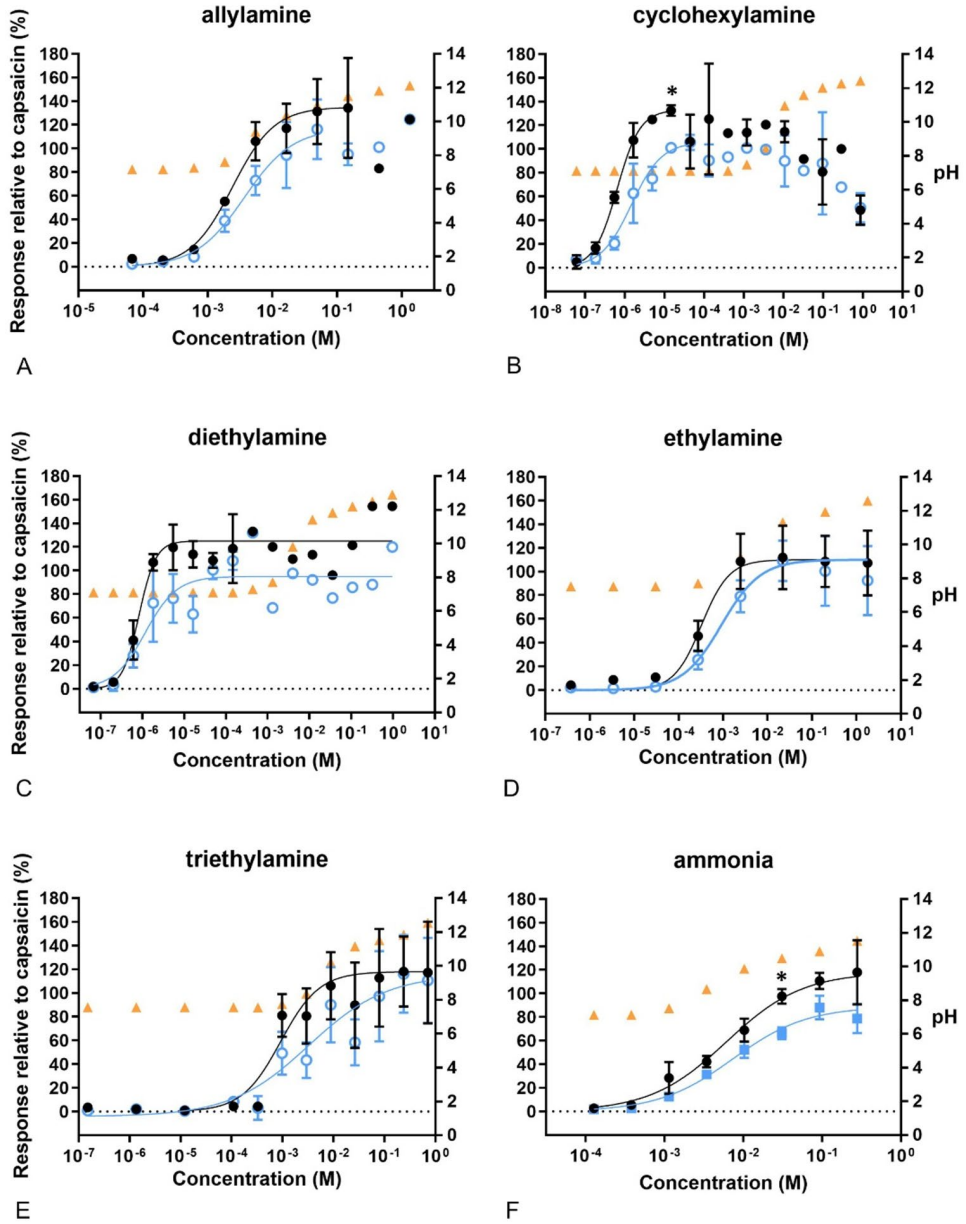
Concentration–response curves of calcium levels measured after exposure to amines. In black, response of the cells to the compounds compared to 100 nM capsaicin. In blue, response of the cells to the compounds after pre-exposure to capsazepine (CZ), related to 100 nM capsaicin. In orange, pH of the final test medium (i.e. the buffer for calcium measurements) at the different concentrations tested. **A** allylamine, **B** cyclohexylamine, **C** diethylamine, **D** ethylamine, **E** triethylamine, **F** ammonia. Data are presented as mean ± SD. *Significantly different from compound +CZ (*p* < 0.05)

**Fig. 8:**
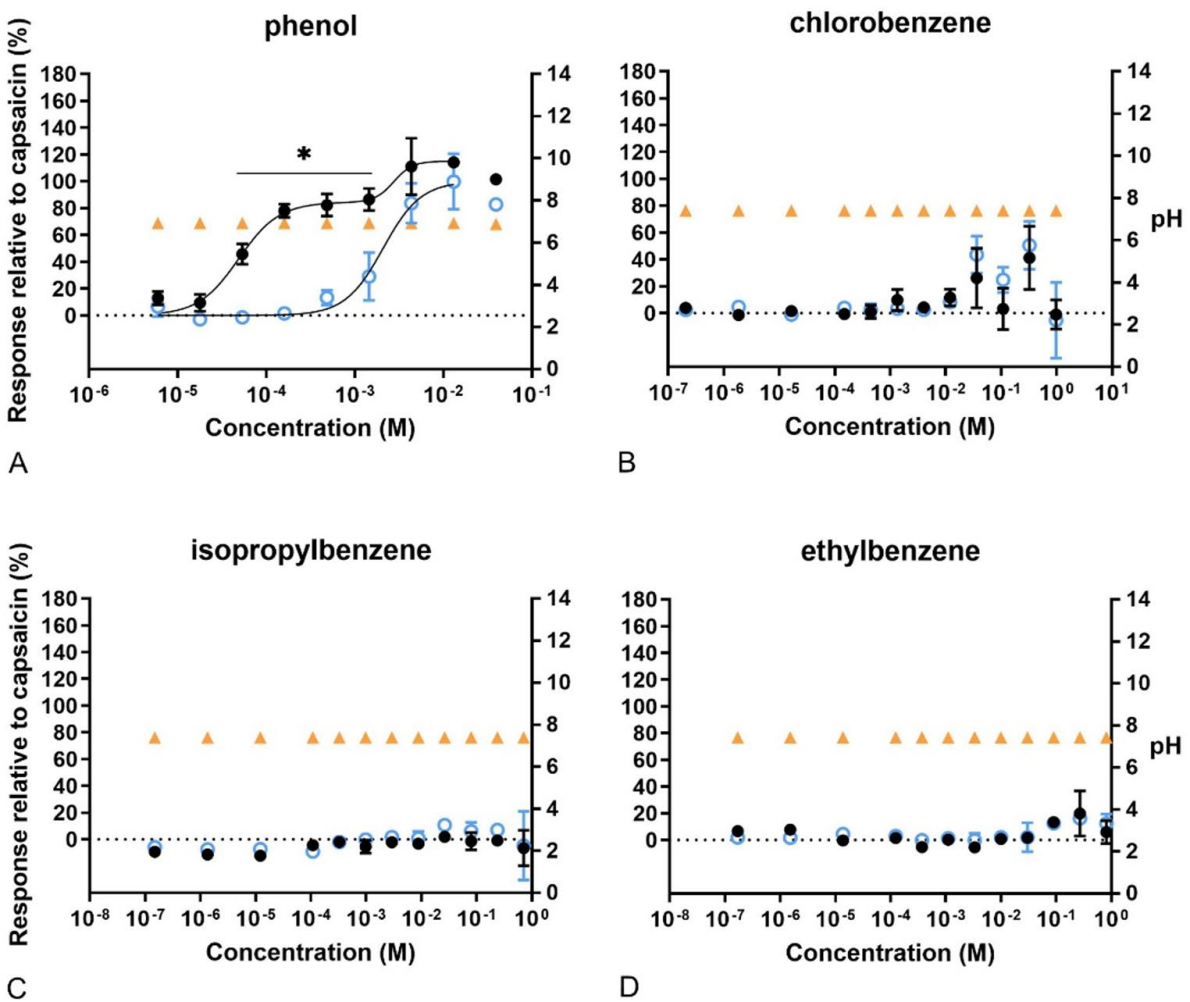
Concentration–response curves of calcium levels measured after exposure to aromatic substances. In black, response of the cells to the compounds compared to 100 nM capsaicin. In blue, response of the cells to the compounds after pre-exposure to capsazepine (CZ), related to 100 nM capsaicin. In orange, pH of the final test medium (i.e. the buffer for calcium measurements) at the different concentrations tested. **A** phenol, **B** chlorobenzene, **C** isopropylbenzene, **D** ethylbenzene. Data are presented as mean ± SD. *Significantly different from compound +CZ (*p* < 0.05)

**Fig. 9:**
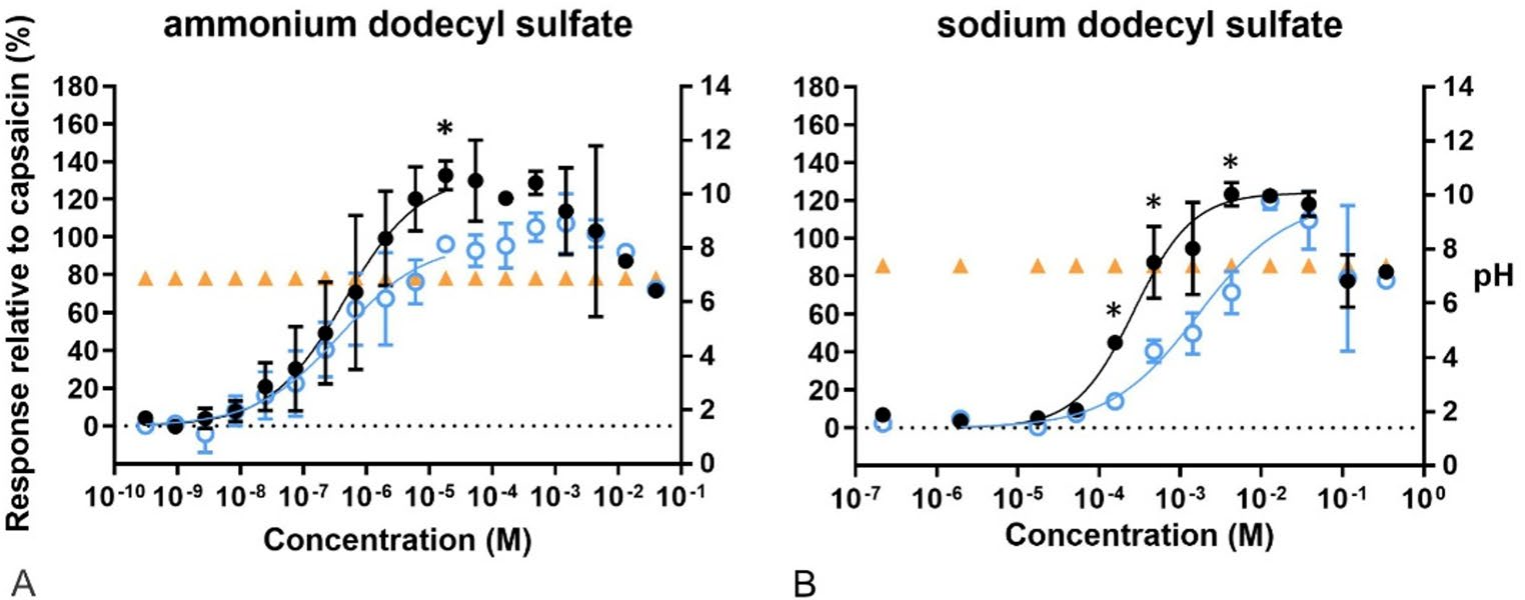
Concentration–response curves of calcium levels measured after exposure to surfactants. In black, response of the cells to the compounds compared to 100 nM capsaicin. In blue, response of the cells to the compounds after pre-exposure to capsazepine (CZ), related to 100 nM capsaicin. In orange, pH of the final test medium (i.e. the buffer for calcium measurements) at the different concentrations tested. **A** Ammonium dodecyl sulfate. **B** Sodium dodecyl sulfate. Data are presented as mean ± SD. *Significantly different from compound +CZ (*p* < 0.05)

**Fig. 10:**
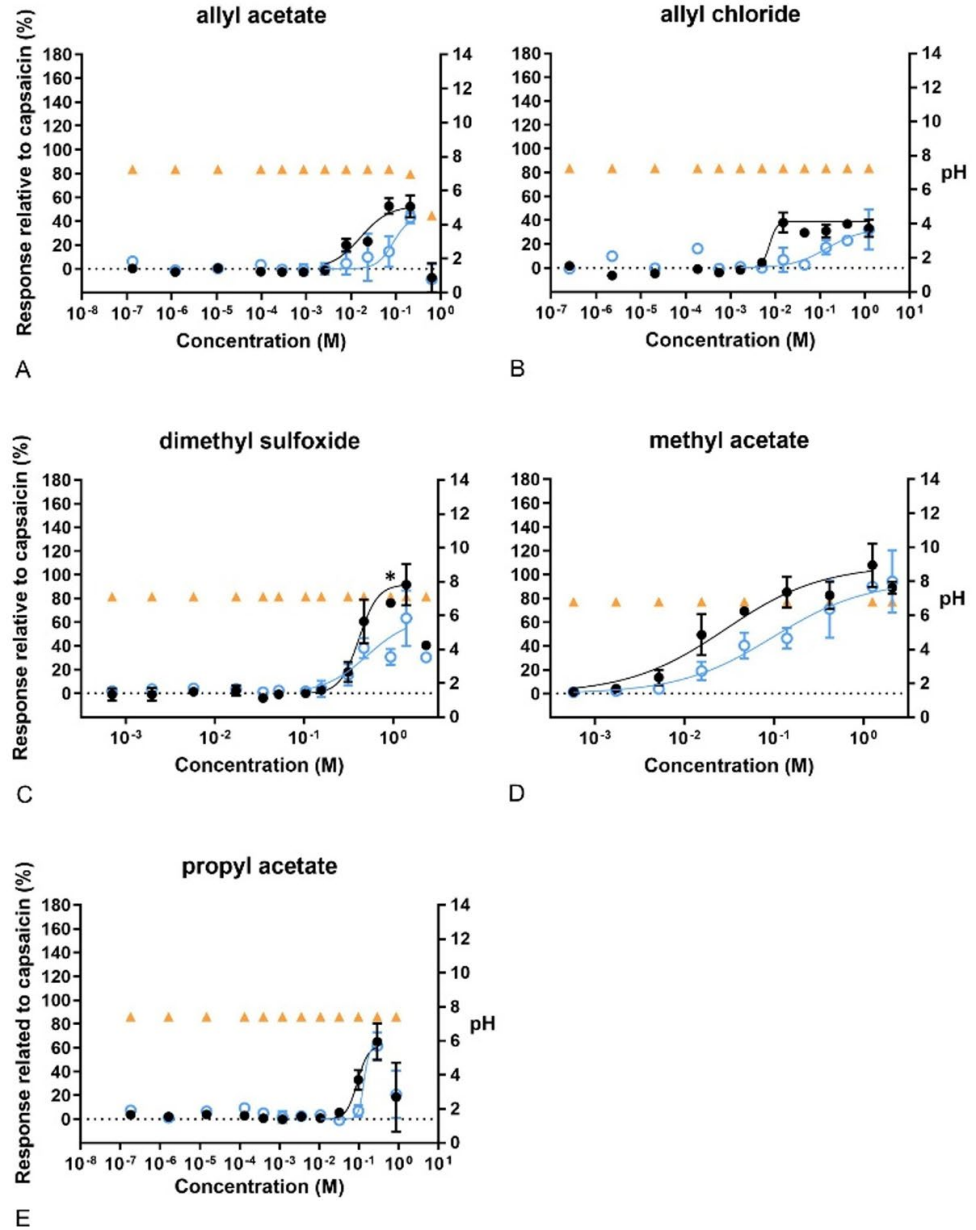
Concentration–response curves of calcium levels measured after exposure to miscellaneous compounds. In black, response of the cells to the compounds compared to 100 nM capsaicin. In blue, response of the cells to the compounds after pre-exposure to capsazepine (CZ), related to 100 nM capsaicin. In orange, pH of the final test medium (i.e. the buffer for calcium measurements) at the different concentrations tested. **A** Allyl acetate, **B** allyl chloride, **C** dimethyl sulfoxide, **D** methyl acetate, **E** propyl acetate. Data are presented as mean ± SD. *Significantly different from compound +CZ (*p* < 0.05)

The cells responded differently to the tested compounds. As can be seen in Fig. 3–10, most of the compounds gave concentration-fluorescence response relationships that followed a sigmoidal curvefit. However, some compounds induced a biphasic response, where the Ca^2+^ influx induced at low concentrations could be blocked by capsazepine. The statistically significant attenuation in the response by capsazepine indicates that some of the compounds acted as TRPV1 agonists. Of the alcohols, the response of 1-propanol at low concentrations (around 0.001 M) could be blocked by capsazepine (Fig. 3). Capsazepine also attenuated the response significantly by methanol at around 1 M. The Ca^2+^ influx induced by the other alcohols could not be blocked by capsazepine. The efficacy (Emax) of the alcohols did not reach 100% of the capsaicin response on Ca^2+^ influx, except for 1-propanol. Furthermore, the fluorescence intensity decreased at the highest concentrations tested of isopropanol (3 M), allyl alcohol (> 0.66 M) and 1-propanol (0.66 M), indicating cytotoxicity. This effect was also observed in the presence of capsazepine. None of the alcohols affected the pH, except allyl alcohol which decreased the pH after exposure to concentrations above 0.32 M.

Concerning the exposure to ketones, 5-methyl-2-hexanone displayed a biphasic response curve with Ca^2+^ influx at 0.0032 M to 0.01 M that could be significantly blocked by capsazepine. The other ketones caused a sigmoidal (monophasic) concentration–response according to the AIC. Nevertheless, the response of low concentrations of 2-hexanone, 4-methyl-2-pentanone, acetone and 2-butanone could be significantly attenuated in the presence of capsazepine, indicating a TRPV1-mediated Ca^2+^ influx (Fig. 4). The Emax of cyclohexanone was 112% of the effect of capsaicin, but none of the other ketones reached an Emax higher than 85% (Table 2). None of the ketones had any effect on the pH in the medium, but cytotoxic concentrations were reached for all compounds, except for acetone and 2-butanone (not shown for 2-hexanone and 2-heptanone for clarity in the graphs).

As expected, butyric acid and acetic acid decreased the pH after the exposure to about 0.001 M and higher concentrations, which led to cytotoxic responses as shown by the decreased fluorescence intensity (Fig. 5). However, butyric acid induced Ca^2+^ influx that could be blocked by capsazepine at concentrations that had no effect on pH. Furthermore, butyric acid was one of the most potent and efficient compounds with EC_20_ equal to 67 nM and Emax at 95% of the capsaicin response (Table 2).

In the case of the aldehydes, both formaldehyde and acetaldehyde gave sigmoidal concentration–response curves (Fig. 6). In addition, capsazepine attenuated Ca^2+^ influx significantly after exposure to some concentrations of formaldehyde. Formaldehyde concentrations above 0.01 M resulted in an increase in pH, while about 0.5 M of acetaldehyde led to a decrease in pH. Again, the acidity correlated with cytotoxicity, shown as a decrease in fluorescence intensity after reaching the Emax for acetaldehyde. In addition, only 1.2 M formaldehyde and 0.59 M acetaldehyde led to a higher response than that obtained for capsaicin.

Exposing the cells to amines resulted in sigmoidal concentration–response curves as well, with Emax values higher than 100% compared to 100 nM capsaicin (Fig. 7). However, capsazepine decreased the Ca^2+^ influx significantly only after exposure to 0.015 mM cyclohexylamine and 31 mM ammonia. Furthermore, all amines caused increased pH at the higher concentrations. Cyclohexylamine and diethylamine, together with ammonium dodecyl sulfate (ADS), showed the highest potency and efficacy of all compounds tested (Table 2 and Supplementary Table S2).

Phenol (Fig. 8) was the only one among the three tested aromatic substances that induced a significantly increased Ca^2+^ influx, the concentration–response curve being biphasic according to the lowest AIC. Notably, the response at low concentrations of phenol was totally blocked by capsazepine, while no effect of the TRPV1 antagonist was observed at the highest concentrations. No significant effects were observed after exposure to chlorobenzene, isopropylbenzene, and ethylbenzene (Fig. 8). Hence, no concentration–response curves could be determined and no in vitro data were obtained for further analyses of these 3 compounds. No change in the buffer pH was observed after addition of the aromatics.

Concerning the exposure to the surfactants ADS and SDS, both compounds led to sigmoidal curvefit responses, but cytotoxicity was observed at the highest concentrations tested as shown by the decrease on fluorescence intensity (Fig. 9). The Ca^2+^ influx caused by exposure to either of compounds was significantly attenuated by capsazepine. Furthermore, the Emax values obtained for both ADS and SDS were higher than the response obtained after exposure to capsaicin (133% and 123%, respectively) and EC20 for ADS (42 nM) was the lowest observed among all compounds tested (Table 2 and Supplementary Table S2). None of the surfactants affected the pH.

Regarding the compounds grouped as “miscellaneous” (Fig. 10), all gave sigmoidal concentration–response curves. None affected the pH, except for the highest concentration of allyl acetate (0.64 M), which resulted in an acidic pH. Only 1.26 M of methyl acetate indicated a higher Emax than that caused by capsaicin. Furthermore, capsazepine attenuated the response of DMSO, which was statistically significant at 0.94 M.

To be able to compare both efficacy and potency of all the compounds without and with TRPV1 blocked by capsazepine, EC20, Emax and conc. at Emax are summarized in Table 2 and with 95% confidence intervals in Supplementary Table S2.

### Principal component analysis

**Fig. 11:**
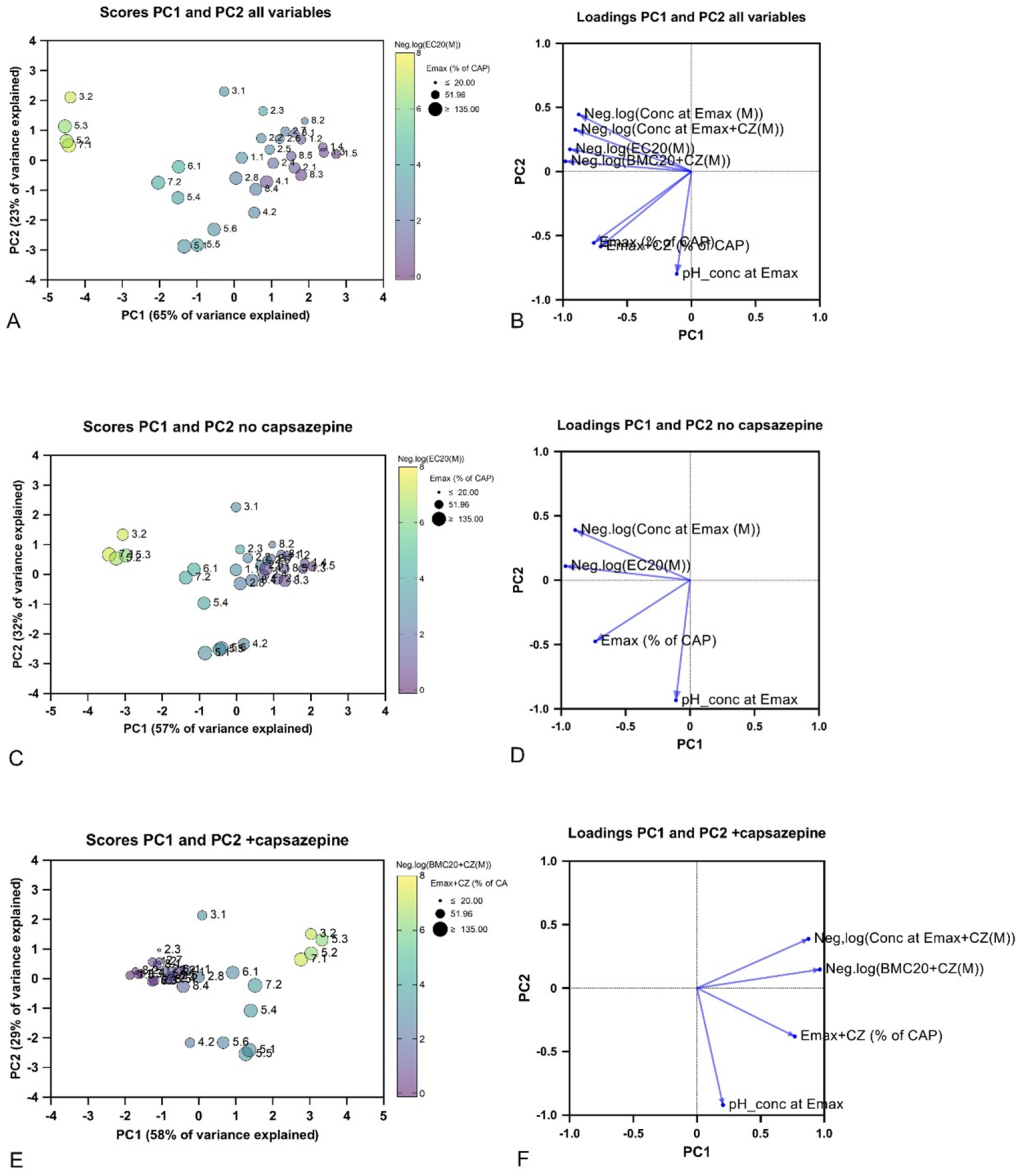
Score and loading plots of the principal component analysis (PCA) of compounds displaying concentration–response of calcium measurements. The variables included are in **A** and **B** negative logarithm of the concentrations giving 20% effect (pEC20 and pEC20 +CZ), maximum Ca2+ influx (Emax and Emax +CZ) and the negative logarithm of concentrations giving maximum effect (p(conc. at Emax) and p(conc. at Emax +CZ)), without and with 30 µM capsazepine (CZ), in **C** and **D**: pEC20, Emax and p(conc. at Emax), and **E** and **F** pEC20 +CZ, Emax +CZ and p(conc. at Emax +CZ)) with 30 µM capsazepine (CZ). pH of the concentration of the compounds at Emax is included in all PCA plots. All effects are normalized to the effect of 100 nM capsaicin. Color scale indicates negative logarithm of EC20 in **A** and **C**, negative logarithm of EC20 +CZ in E, size indicates Emax

Three PCAs were performed with variables for EC20, Emax, the concentration at Emax and the pH at Emax for the compounds that generated dose–response curves (i.e. all but chlorobenzene, isopropylbenzene, and ethylbenzene). One PCA included variables without capsazepine, measuring possible effects mediated by TRPV1 activation (Fig. 11B), one PCA included variables obtained in the presence of capsazepine, measuring non-TRPV1-mediated Ca^2+^ influx (Fig. 11C) and one PCA included all variables obtained from experiments without and with capsazepine (Fig. 11A). Principal component 1 (PC1) explained 65%, 57% and 58% of the variance in Fig. 11A–C, respectively. The concentration variables, indicating potency, contributed with similar loadings to PC1, whereas Emax (efficacy) and particularly pH contributed more than the other variables to PC2 (Fig. 11B, D, F). Together, PC1 and PC2 explained 88%, 89% and 87% of the variance in Fig. 11A–C, respectively. The PCA including all variables diverged the scores of the compounds slightly more (Fig. 11A), but it was clear that the most irritating compounds clustered in all 3 PCAs, with butyric acid, ADS, cyclohexylamine, and diethylamine displaying the most distant scores from 0 in PC1. Interestingly, the most irritating chemicals (RD_50_ lower than 700 ppm, Tables 4, 5) were separated from the rest at PC1 around 0, indicating that the potency had a slightly higher impact than the efficacy on irritancy and that pH had minor impact, considering loadings for PC1 (Fig. 11).

**Table 5:**
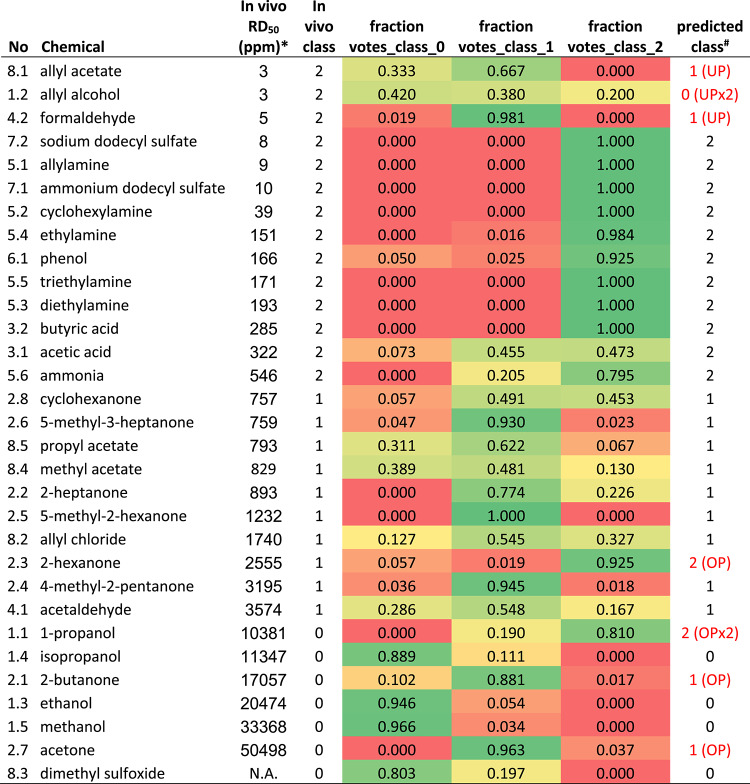
Probability for classification into the 3-class model by including TRPV1 in the in vitro variables (No CZ) in the random forest prediction. Heat map indicates fraction of votes in each class (colour figure online)

^*^Average RD_50_ of values reported by Schaper (1993)

^#^UP; underpredicted by 1 class, UPx2; underpredicted by 2 classes, OP; overpredicted by 1 class, OPx2; overpredicted by 2 classes

N.A., not available

Colours indicate the fraction of votes, being in green equal o similar to 1, and in red equal or similar to 0

### Machine learning (random forest) analysis

The combinations of variables that were included in the 3 PCA analyses, together with PC1 and PC2 from each PCA, were used to predict respiratory irritation classification in 6 models, based on average RD_50_ values from literature. The results from the machine learning, random forest, analyses are shown in Table 3. The 2-class (binary) classification show that the +CZ descriptors (blocking TRPV1) achieved the highest overall accuracy of 0.90 over all 100 test sets while the No CZ descriptors (including possible TRPV1 activation) showed the lowest accuracy of 0.81. When including all variables (all_invitro, including both +CZ and No CZ variables), the overall accuracy was 0.87. For the corresponding 3-class prediction, the overall accuracy of all_invitro as well as +CZ was 0.71 for both. For No CZ the overall accuracy was slightly higher, 0.77. The outlier predictions were low with only 7.1, 4.8 and 5.7 percent, for all_in vitro, +CZ and No CZ, respectively, in the 2-class model. No CZ, considering possible TRPV1-mediated Ca^2+^ influx, had an outlier percentage of 5.7, +CZ (blocking TRPV1) 4.8% and all_invitro 7.1% in the 3-class model (Table 3). It must be emphasized, though, that no statistical difference was observed between the different random forest models and the conclusions about the best performing model should only be indicative.

When looking at the fraction of the 100 votes for each compound, the most irritating compounds with lowest RD_50_ values, i.e. allyl acetate, allyl alcohol, and formaldehyde, were incorrectly predicted as intermediately/not irritating in the 2-class and the 3-class models (Tables 4, 5). However, all 3 compounds are severe irritants in vivo with average RD_50_ values of 3, 5 and 3 ppm, respectively. Four other compounds were misclassified in the best performing, No CZ in vitro*,* 3-class model and were predicted as more irritating than determined by the in vivo assay. The compound 2-hexanone was classified as a severely irritating compound in the 3-class model, but is an intermediately irritating compound according to the average RD_50_ value (2555 ppm). In the 2-class model the fraction of votes for 2-hexanone was almost equally distributed between intermediately irritating/non-irritating (0.528) and severely irritating (0.472) classes (Table 4). Butanone and acetone were predicted to be intermediately irritating, but are not irritating in vivo. 1-propanol, which displayed a bi-phasic response and a significant agonistic effect on TRPV1, was classified as a severe irritant in the cell model, but a non-irritant (class 0) according to the average RD_50_ value (Table 5). Tables for 2-class models of All_invitro and No CZ and 3-class models for +CZ and All_in vitro are presented in Supplementary Tables 4–7, respectively.

## Discussion

### *Mode of action of chemically induced Ca*^*2*+^*influx in the TRPV1-SH-SY5Y cell model*

Chemically induced irritation in the upper respiratory tract can cause significant health problems (Brüning et al. 2014). Hence, OELs should be determined to set acceptable limits. This can be done by exposing human volunteers to fixed doses or by using the RD_50_ test in rodents. To circumvent ethical issues with human and animal exposure, we evaluated the possibility to use a neuronal cell line with stable expression of a nociceptor that is mediating neurosensory irritation. We found that chemicals from different chemical groups displayed a wide range of potency and efficacy but no chemical was as potent as capsaicin, the principle respiratory irritant/positive control of sensory irritation. Ammonium dodecyl sulfate, butyric acid, cyclohexylamine, and diethylamine were the most potent and efficient compounds with EC_20_ values in the nanomolar range and Emax over 100% of the response of capsaicin, or just below. The effects of ADS and SDS agree with a previous study, indicating that surfactants are TRPV1 agonists (Lilja et al. 2007; Lindegren et al. 2009). According to Frank et al. (2018), TRPV1 may be a principal receptor mediating pungency of some detergents and soaps by binding to the aliphatic part of these compounds, as anionic aliphatic surfactants have shown to be strong TRPV1 agonists (Frank et al. 2018).

The least potent and efficient, water soluble, chemicals were methanol, ethanol, and isopropanol, all having EC_20_ values in the molar range and Emax around 50–60%. It has previously been shown that alcohols can activate and sensitize the TRPV1 channel (Trevizani et al. 2002; Frank et al. 2018) but only 1-propanol, methanol, and phenol were indicated as significant TRPV1 agonists in our study. The full blockage of phenol-induced Ca^2+^ influx by capsazepine was unexpected and a novel finding, as well as the significant TRPV1-specific response of 1-propanol at the lower concentrations tested. Both compounds displayed a biphasic concentration-fluorescence response as determined by the lowest AIC in comparison to sigmoidal curve fit, which further suggests that these compounds are TRPV1 agonists at low concentrations. The weak response by the primary alcohols on Ca^2+^ influx is in line with a previous report, indicating that 1 mM had only a slight effect on cells transfected with hTRPV1 (Komatsu et al. 2012). Nevertheless, the same study showed that 1 mM of the same compounds induced an increase in the intracellular Ca^2+^ concentration in cells expressing hTRPA1. However, patch-clamp analysis on the hTRPA1-expressing cells indicated that ethanol did not evoke current responses in a range of 0.1–100 mM ethanol, suggesting that the increase in Ca^2+^ influx observed in hTRPA1-expressing cells at the higher concentration was due to an effect that is unrelated to TRPA1 or TRPV1 activation (Komatsu et al. 2012). When we treated our cells with the ethanol metabolite acetaldehyde, a response unrelated to TRPV1 activation was obtained, as there was an increase in the Ca^2+^ influx after exposure to some concentrations, but the response was not prevented in presence of the antagonist capsazepine. Bang et al. (2007) tested 1 mM acetaldehyde in hTRPA1-expressing cells, detecting a rapid increase in intracellular Ca^2+^ levels upon extracellular addition, while non-transfected cells did not show any changes up to 10 mM. The EC_50_ value for acetaldehyde in hTRPA1 expressing cells was 76.5 µM, which is about three magnitudes lower than the EC_20_ value of 0.14 M, determined in the TRPV1-SH-SY5Y cell model. Bang et al. also performed whole-cell voltage-clamp experiments, detecting a pronounced outward rectifying current during 1 mM acetaldehyde application in mTRPA1 cells, with an EC_50_ of 1.19 mM, which was suppressed in the presence of the TRPA1 antagonist camphor (Bang et al. 2007). No effects were observed when acetaldehyde was applied to cells expressing TRPV1 among other TRP channels (except TRPA1). These findings strongly indicate that acetaldehyde is a TRPA1 agonist, which can explain the moderate potency in our cell model.

Exposure to formaldehyde, the other aldehyde tested in the present study, resulted in a significant increase in Ca^2+^ influx, but the response was only partially blocked with capsazepine, indicating that TRPV1 activation is not the only mode of action involved. Formalin (in which formaldehyde is the active ingredient) is the principal agent used in an in vivo model developed more than 30 years ago to assess chemically induced pain and to evaluate analgesic drugs in laboratory animals. McNamara et al. (2007) used a formalin solution to generate Ca^2+^ influx in TRPA1-transfected cells. Neither the native (non-transfected) cells, nor cells expressing TRPV1, TRPV2 or TRPV3 responded to the same concentrations of formalin. EC50 values determined from the responses in hTRPA1 and in rTRPA1 were 0.0016% and 0.0015%, respectively (McNamara et al. 2007). These concentrations correspond to approximately 0.200 mM, a concentration that did not result in a significant response in our model. The same authors also used whole-cell patch-clamp to assess formalin-evoked membrane currents in hTRPA1-expressing cells, which led to outwardly rectifying currents that were attenuated by a non-selective TRP channel inhibitor (McNamara et al. 2007). Thus, formaldehyde would be an agonist for TRPA1 receptor, but not for TRPV1. However, capsazepine attenuated the efficacy of formaldehyde induced Ca^2+^ influx in our study, indicating that the compound is a TRPV1 agonist, but this effect occurred at concentrations that caused an increase in the pH. Thus, the mode of action in our cell model is unclear.

The TRPV1 channel is pH sensitive and can be activated by low pH stimuli (Caterina et al. 1997). The receptor is therefore mediating nociception caused by acidification, which can be measured in the pain behavior model of acetic acid-induced writhing (Andreev et al. 2013). Interestingly, the pH seemed to have only minor effect on the Ca^2+^ influx, which was surprising since the SH-SY5Y cells express acid sensing ion channels (ASICs) natively (Xiong et al. 2012). In fact, Silver et al. (2006) also reported that the application of acetic acid at pH 4.4 in both TRPV1-transfected and non-transfected cells resulted in Ca^2+^ influx. The effect of acids has also been studied on TRPA1 activity, which seems to be a more specific target for low pH than TRPV1. Kumar et al. (2021) exposed TRPA1 expressing STC-1 cells to acetic acid (1, 5, 10 mM) and butyric acid (1, 2.5, 5 mM), which resulted in dose-dependent intracellular Ca^2+^ influx for both compounds. The responses were attenuated in the presence of a non-selective TRP channel inhibitor. In the same study, the selective TRPA1 antagonist HC-030031 prevented the elevation in intracellular Ca^2+^ levels at all doses of acetic acid, but a significant effect was observed only after exposure to 5 mM butyric acid (Kumar et al. 2021). Interestingly, butyric acid was one of the most potent compounds tested in our assay, also indicating TRPV1 agonism at sub-micromolar concentrations that did not have any effect on pH. Wang et al. (2011) reported that acetic acid at pH 5 induced patch-clamp recorded currents that were strong and rapid in rTRPA1 expressing cells, which was larger than the currents obtained after exposure to a HCl solution with the same pH. This indicates that the effect of acetic acid could not be attributed to protons affecting the receptor. These authors also reported that acetic acid activated large currents in a subset of cells transfected with hTRPA1 and mTRPA1, indicating that activation of the channel by acetic acid may lead to irritant effects of weak acids in humans as well as in rodents (Wang et al. 2011).

All of the ketones tested in our study gave responses in Ca^2+^ influx. Exposure to 2-butanone, 2-hexanone, 4-methyl-2-pentanone, 5-methyl-2-hexanone and acetone resulted in significant differences between presence and absence of capsazepine, suggesting TRPV1 mediated mode of activation. Exposure to 2-heptanone, 5-methyl-3-heptanone and cyclohexanone led to responses that were not significantly prevented by capsazepine. In this sense, Saunders et al. (2013) reported that cyclohexanone and capsaicin led to significant less aversion in TRPV1 knockout mice compared to the wildtype in the cotton swab test. Furthermore, in the same experiments, cyclohexanone depressed the respiration rate of wildtype mice in a dose-dependent manner (1200, 1600, 2000, 3000 ppm), while the respiration rate was not altered from its baseline in TRPV1 knockout mice at any concentration (Saunders et al. 2013). The high doses can be questioned, though, since the reported average RD_50_ value for mouse, used as in vivo reference in our study, is around 750 ppm (Schaper 1993). Unspecific tissue damage may release inflammatory factors that can activate TRPV1 in vivo (reviewed by Shuba 2021). Hence, TRPV1 activation in vivo may not be the primary mode of action of compounds that induce sensory irritation, although trpv1 knock out animals and TRPV1 antagonists abolish chemically induced responses.

Amines are reactive and are believed to have an unspecific mode of action. According to the RD_50_ values, unsaturated amines are more potent than saturated amines, with allyl amine being the most potent amine included in our study (Alarie et al. 1998). All amines were correctly classified as irritants in our study, but judging by the EC_20_ values (Table 2), the ranking differed in that the saturated cyclohexylamine was the most potent and effective amine, followed by the diethyl-, ethyl- and triethylamines. Allyl amine was less potent but possessed as high efficacy as cyclohexylamine. The Ca^2+^ influx induced by cyclohexylamine was clearly blocked by capsazepine, indicating that cyclohexylamine may have agonistic effects on TRPV1.

Dimethyl sulfoxide led to Ca^2+^ influx in our cell model. In this case, only the concentration close to 1M was partially prevented in the presence of capsazepine. In this regard, Ivanova et al. (2024) tested 10, 30, and 50% DMSO solutions injected intraperitoneally (10 mL/kg) 60 min before administration of capsaicin solution, which did not result in intensification, but conversely, a dose-dependently reduction of nociception in mice caused by activation of TRPV1.

The benzene derivatives displayed no significant effect in our cell system and could not be included in further analyses. The lack of effect in vitro may be correlated to their low solubility in water, i.e. Log K_ow_ values > 2.5 (PubChem). Hence, the applicability domain of this cell assay excludes extremely non-polar compounds, which is a common disadvantage of assays that are based on cells in regular culture media. For chemicals that cannot be tested in aqueous solutions or are highly volatile, physicochemical properties may be an alternative strategy to use for classification of respiratory irritancy (Alarie et al. 1998).

### Correlation of the in vitro-derived data with historical in vivo data

The 2-class (binary) models + CZ gave the best accuracy in the prediction of irritating compounds and intermediately/non-irritating compounds. In the 3-class models, including an intermediately irritating class with RD_50_ between 700 and 10,000 ppm as a separate class, variables for TRPV1 activation (No CZ) displayed a slightly better accuracy than variables excluding TRPV1 activation. These results may indicate that the most severe respiratory irritation is mediated by mechanisms that do not include TRPV1 activation, although intermediate irritation may have a TRPV1 mediated mode of action. The reason why some compounds were classified as more irritating in the No CZ model in comparison to the RD_50_ values may be that TRPV1 activation with low efficacy in vitro may not be sufficient to induce 50% respiratory depression in mice. The TRPV1 activation in vitro will, when using RD_50_ values as reference, overpredict respiratory irritation and the chemicals displaying agonist effects will be misclassified. This was probably the reason why 2-hexanone, 1-propanol, 2-butanone and acetone were predicted to be more irritating by the in vitro model than by the RD_50_ test.

The machine learning analysis misclassified the 3 most irritating compounds with RD_50_ < 5 ppm, i.e. formaldehyde, allyl acetate and allyl alcohol. The high reactivity of formaldehyde results in covalent bonds with nucleophilic moieties, e.g. in proteins (Alarie et al. 1998). One reason for the misclassification of formaldehyde, allyl acetate and allyl alcohol may be that the TRPV1-SH-SY5Y cell model does not express TRPA1 channels, which are able to sense a wide array of irritants through covalent and allosteric binding events (Stenger et al. 2015; Zsidó et al. 2021). Classical agonists of TRPA1 are acrolein, allicin, aldehydes (including formaldehyde), isocyanates and tear gas compounds (Martinez and Eling 2019). One major mechanism of TRPA1 activation involves oxidation of cysteine residues in the ankyrin repeats of TRPA1’s cytoplasmic domain. This reactivity-based sensing mechanism is believed to account for the diverse range of TRPA1 agonists (Frank et al. 2018), which indeed, may be the mode of action for the 3 chemicals that were significantly under-predicted by the TRPV1-SH-SY5Y cell model in terms of respiratory irritation. Nevertheless, the TRPA1 agonists acetaldehyde (Bang et al. 2007) and ethanol (Komatsu et al. 2012) were correctly predicted to be intermediately irritating and non-irritating compounds, respectively, by the TRPV1-SH-SY5Y in vitro model. In this regard, the exposure to irritants that cause the activation of TRPA1 and/or TRPV1 can lead to clinical responses in the organism such as chronic cough, pain, airway inflammation, COPD and asthmatic like conditions (Bessac and Jordt 2008; Baraldi et al. 2010; Chen and Hackos 2015). Activation of the TRPA1 has been proposed to be a molecular initiating event for respiratory irritation caused by volatile compounds (Martinez and Eling 2019). Nevertheless, it has been shown that TRPV1 channels are additional primary targets for sensory irritation (Bessac and Jordt 2008; Lehmann et al. 2017) and may also positively interact with TRPA1 in bronchopulmonary sensory neurons (Hsu and Lee 2015). Our study shows that TRPV1 may play a role as the target for molecular initiating event, especially for intermediately irritating compounds. Hence, we believe that our in vitro and in silico model would fit in a battery of tests for respiratory sensory irritation, together with assays that can detect TRPA1 channel activation.

## Conclusion

We show that Ca^2+^ measurements in the TRPV1-SH-SY5Y assay can predict severe respiratory irritation with the exception of chemicals that induce sensory irritation by a specific mode of action that cannot be assessed in the model. This mode of action is most probably by TRPA1 activation. The Ca^2+^ influx caused by several of the tested chemicals was attenuated in the presence of capsazepine, indicating that organic compounds and surfactants affect TRPV1 permeability with phenol as a putative agonist. The machine learning, random forest analyses show that severe respiratory irritancy is not dependent on TRPV1 activation, but may be involved in intermediate/moderate respiratory irritation when correlated to in vivo RD_50_ values in ppm. However, the TRPV1 mediated response seemed to over-predict irritancy somewhat, placing 4 compounds in classes that predicted more irritation than the in vivo test. We suggest that the TRPV1-SH-SY5Y cell model, together with an assay that covers TRPA1 activation, combined with machine learning models, may be useful for classification of respiratory irritation.

## Supplementary Information

Below is the link to the electronic supplementary material.

Supplementary Material 1

## Data Availability

The data will be available upon request.
