# Supplementary material for: Classification of industrial chemicals for respiratory chemosensory irritation using the TRPV1-expressing neuronal SH-SY5Y cell model and machine learning
Source: Arch Toxicol. 2026 Jan 22;100(4):1301–20. doi: 10.1007/s00204-025-04288-6 (PMC13043554; doi:10.1007/s00204-025-04288-6)
Supplement: Supplementary file 1 — Supplementary Material 1 [file 204_2025_4288_MOESM1_ESM.docx]

**Supplementary files**

**Classification of industrial chemicals for respiratory chemosensory irritation with the TRPV1-expressing neuronal SH-SY5Y cell model**

**María Hinojosa^1,2^, Gunnar Johanson^2^, Ulf Norinder^3,4^, and Anna Forsby^1^**

**^1^Department of Biochemistry and Biophysics, Stockholm University, Stockholm, Sweden**

**^2^Institute of Environmental medicine, Karolinska Institute, Stockholm, Sweden**

**^3^ MTM Research Centre, School of Science and Technology, Örebro University, Örebro, Sweden**

**^4^ Department of Pharmaceutical Biosciences, Uppsala University, Uppsala, Sweden**

**Index**

[Supplementary Table S1. Suppliers and reference numbers of chemicals and material 3](#_Toc192085253)

[Supplementary Figure S1. Example of SoftMax registrations 5](#_Toc192085254)

[Supplementary Table S2. EC20 with 95%CI 6](#_Toc192085255)

[Supplementary Table S3. Data for all variables used in the 6 prediction models. 8](#_Toc192085256)

[Supplementary Table S4. Probability for classification into the 2-class model by using all in vitro variables (no CZ and +CZ) in the random forest prediction 10](#_Toc192085257)

[Supplementary Table S5. Probability for classification into the 2-class model by using in vitro variables for no CZ in the random forest prediction 11](#_Toc192085258)

[Supplementary Table S6. Probability for classification into the 3-class model by using all in vitro variables (no CZ and +CZ) in the random forest prediction. 12](#_Toc192085259)

[Supplementary Table S7. Probability for classification into the 3-class model by using in vitro variables for +CZ in the random forest prediction. 13](#_Toc192085260)

### Supplementary Table S1. Suppliers and reference numbers of chemicals and material

| **Chemical** | **CAS number** | **Reference** | **Supplier** |
| --- | --- | --- | --- |
| Capsaicin | 404-86-4 | M2028 | Sigma-Aldrich |
| Capsazepine | 138977-28-3 | C191 | Sigma-Aldrich |
| Fura-2AM | 108964-32-5 | 47989 | Sigma-Aldrich |
| D-glucose | 50-99-7 | G7021 | Sigma-Aldrich |
| HEPES | 7365-45-9 | H3375 | Sigma-Aldrich |
| NaCl | 7647-14-5 | S5886 | Sigma-Aldrich |
| KCl | 7447-40-7 | P3911 | Sigma-Aldrich |
| MgSO4x7H2O | 10034-99-8 | M2773 | Sigma-Aldrich |
| KH_2_PO_4_ | 7778-77-0 | P5655 | Sigma-Aldrich |
| CaCl_2_x2H_2_O | 10035-04-8 | C7902 | Sigma-Aldrich |
| Puromycin dihydro chloride | 58-58-2 | P8833 | Sigma-Aldrich |
| 1-propanol | 71-23-8 | 71238 | Sigma-Aldrich |
| 2-butanone | 78-93-3 | 2011590 | Sigma-Aldrich |
| 2-heptanone | 110-43-0 | 2037671 | Sigma-Aldrich |
| 2-hexanone | 591-78-6 | 591786 | Sigma-Aldrich |
| 4-methyl-2-pentanone | 108-10-1 | 2035501 | Sigma-Aldrich |
| 5-methyl-2-hexanone | 110-12-3 | 110123 | Sigma-Aldrich |
| 5-methyl-3-heptanone | 541-85-5 | 541855 | Sigma-Aldrich |
| Acetaldehyde | 75-07-0 | 75070 | Sigma-Aldrich |
| Acetic acid | 64-19-7 | 64197 | Sigma-Aldrich |
| Acetone | 67-64-1 | 2006622 | Sigma-Aldrich |
| Allyl acetate | 591-87-7 | 591877 | Sigma-Aldrich |
| Allyl alcohol | 107-18-6 | 2034707 | Sigma-Aldrich |
| Allyl chloride | 107-05-1 | 2034576 | Sigma-Aldrich |
| Allylamine | 107-11-9 | 241075 | Sigma-Aldrich |
| Ammonium dodecyl sulfate solution (ADS) | 2235-54-3 | 681806 | Sigma-Aldrich |
| Benzyl chloride | 100-44-7 | 100447 | Sigma-Aldrich |
| Butyric acid | 107-92-6 | 107926 | Sigma-Aldrich |
| Chlorobenzene | 108-90-7 | 2036285 | Sigma-Aldrich |
| Cyclohexanone | 108-94-1 | 2036311 | Sigma-Aldrich |
| Cyclohexylamine | 108-91-8 | 108918 | Sigma-Aldrich |
| Diethylamine | 109-89-7 | 2037163 | Sigma-Aldrich |
| Diisopropylamine | 108-18-9 | 2035585 | Sigma-Aldrich |
| Dimethyl sulfoxide (DMSO) | 67-68-5 | 67685 | Sigma-Aldrich |
| Ethanol | 64-17-5 | 64175 | Sigma-Aldrich |
| Ethylamine | 75-04-7 | 75047 | Sigma-Aldrich |
| Ethylbenzene | 100-41-4 | 100414 | Sigma-Aldrich |
| Formaldehyde | 50-00-0 | 50000 | Sigma-Aldrich |
| Furfural | 98-01-1 | 2026277 | Sigma-Aldrich |
| Isopropanol | 67-63-0 | 67630 | Sigma-Aldrich |
| Isopropylbenzene | 98-82-8 | 98828 | Sigma-Aldrich |
| Methanol | 67-56-1 | 2006596 | Sigma-Aldrich |
| Methyl acetate | 79-20-9 | 79209 | Sigma-Aldrich |
| Phenol | 108-95-2 | 2036327 | Sigma-Aldrich |
| Propyl acetate | 109-60-4 | 109604 | Sigma-Aldrich |
| Sodium dodecyl sulfate (SDS) | 151-21-3 | 151213 | Sigma-Aldrich |
| Triethylamine | 121-44-8 | 121448 | Sigma-Aldrich |
| **Product** |  |  |  |
| Advanced minimal essential medium (AdvMEM) |  | 12492-013 | Gibco |
| Fetal bovine serum, standard sterile filtered |  | 10270-106 | Gibco |
| Penicillin/streptomycin |  | 15140-122 | Gibco |
| L-glutamine |  | 25030-024 | Gibco |
| Non-essential amino acids |  | 11140-035 | Gibco |
| TrypLE Express Enzyme |  | 12605010 | Gibco |
| Cell culture flasks 75 cm^2^ |  | 430641 | Corning |
| Clear culture 96-well plates with clear bottom |  | 3599 | Corning |
| 96-well standard plates with flat bottom |  | 3364 | Corning |
| Universal optical sealing tape |  | 6575 | Corning |
| 50 mL reagent reservoirs |  | 4870 | Corning |
| disposable reservoirs liner, 8-channel |  | 4878 | Corning |
| Cryo tubes 2 mL |  | 431386 | Corning |
| 1.5 mL glass vials |  | 548-0028 | VWR |
| Black pipette tips 200 µL |  | 9000-0911 | Molecular Devices |

### Supplementary Figure S1. Example of SoftMax registrations

Example of SoftMax registrations of fluorescence intensity (FI) ratios between 340 nm and 380 nm excitation wavelengths, indicating Ca^2+^-bound and free Fura2 fluorescence, respectively. Each curve represents the baseline FI before addition (0-30 seconds) and the change in FI after addition of capsaicin alone in the presence of the TRPV1 channel antagonist 30 µM capsazepine (30-180 seconds) in one well in a 96-well plate.


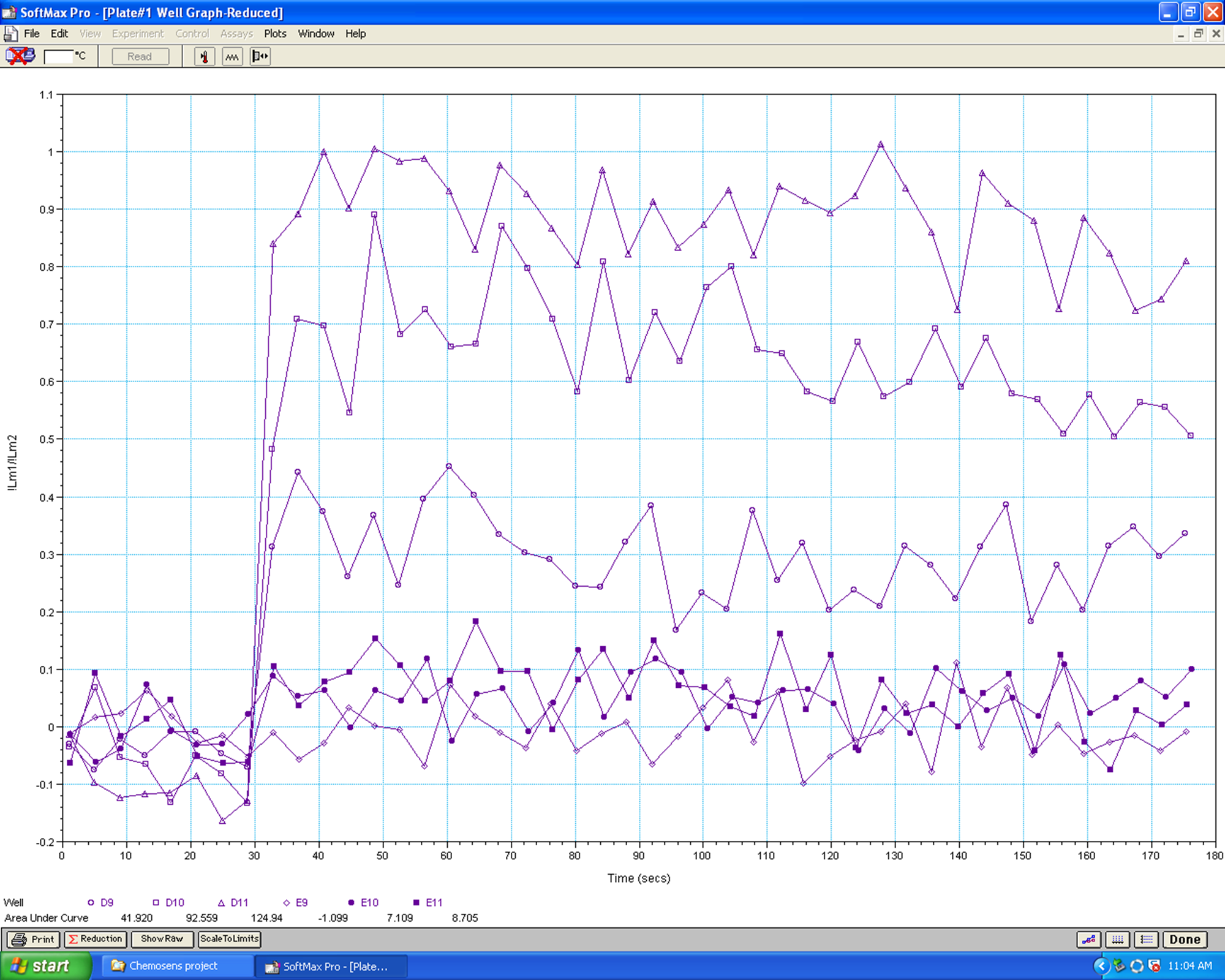


100 nM capsaicin

10 nM capsaicin

1 nM capsaicin

100 nM capsaicin
 + 30 µM CZ

10 nM capsaicin

+ 30 µM CZ

1 nM Capsaicin

+ 30 µM CZ

### Supplementary Table S2. EC20 with 95%CI

Concentrations of the compounds giving 20% increase in calcium influx (EC20) in relation to 100 nM capsaicin with upper and lower EC20 at 95% confidence interval (from curve fit equations used in Figures 2-10 in the manuscript).

|  | EC20 (M) | Upper EC20 (M) | Lower EC20 (M) |
| --- | --- | --- | --- |
| **Capsaicin** | 4.9E-10 | 6.3E-10 | 3.6E-10 |
| **1. Alcohols** |  |  |  |
| 1.1. 1-propanol | 5.0E-04 | ambigous | ambigous |
| 1.2. allyl alcohol | 6.8E-02 | 1.1E-01 | 3.4E-02 |
| 1.3. ethanol | 2.2E-01 | 2.9E-01 | 1.5E-01 |
| 1.4. isopropanol | 1.4E-01 | 1.9E-01 | 9.4E-02 |
| 1.5. methanol | 5.6E-01 | ambigous | ambigous |
| **2. Ketones** |  |  |  |
| 2.1. 2-butanone | 4.1E-02 | 6.7E-02 | 2.2E-02 |
| 2.2. 2-heptanone | 1.2E-03 | 4.4E-03 | am |
| 2.3. 2-hexanone | 9.6E-05 | 3.3E-04 | 1.4E-05 |
| 2.4. 4-methyl-2-pentanone | 1.5E-02 | 2.6E-02 | 6.9E-03 |
| 2.5. 5-methyl-2-hexanone | 1.2E-03 | ambigous | ambigous |
| 2.6. 5-methyl-3-heptanone | 1.9E-03 | 6.7E-03 | 2.5E-04 |
| 2.7. acetone | 6.5E-03 | 9.5E-03 | 4.1E-03 |
| 2.8. cyclohexanone | 4.1E-03 | 5.9E-03 | 2.7E-03 |
| **3. Acids** |  |  |  |
| 3.1. acetic acid | 6.5E-04 | ND | 4.4E-04 |
| 3.2. butyric acid | 6.7E-08 | ambigous | ambigous |
| **4. Aldehydes** |  |  |  |
| 4.1. acetaldehyde | 1.4E-01 | 1.6E-01 | 1.2E-01 |
| 4.2. formaldehyde | 2.4E-03 | 4.0E-03 | 1.5E-03 |
| **5. Amines** |  |  |  |
| 5.1. allylamine | 6.6E-04 | 1.2E-03 | 3.5E-04 |
| 5.2. cyclohexylamine | 2.0E-07 | 2.6E-07 | 1.6E-07 |
| 5.3. diethylamine | 4.4E-07 | 6.1E-07 | 2.3E-07 |
| 5.4. ethylamine | 1.1E-04 | 2.2E-04 | 4.2E-05 |
| 5.5. triethylamine | 2.3E-04 | 6.7E-04 | 6.1E-05 |
| 5.6. ammonia | 1.0E-03 | 1.7E-03 | 6.2E-04 |
| **6. Aromatic substances** |  |  |  |
| 6.1. phenol | 2.7E-05 | 3.7E-05 | 1.7E-05 |
| 6.2. chlorobenzene | NE |  |  |
| 6.3. isopropylbenzene | NE |  |  |
| 6.4. ethylbenzene | NE |  |  |
| **7. Surfactants** |  |  |  |
| 7.1. ammonium dodecyl sulfate | 4.2E-08 | 9.4E-08 | 1.7E-08 |
| 7.2. sodium dodecyl sulfate | 6.9E-05 | 1.1E-04 | 4.1E-05 |
| **8. Miscellanous** |  |  |  |
| 8.1. allyl acetate | 1.1E-02 | 1.9E-02 | 6.3E-03 |
| 8.2. allyl chloride | 7.6E-03 | 1.3E-02 | 5.3E-03 |
| 8.3. dimethyl sulfoxide | 3.0E-01 | 3.6E-01 | 2.4E-01 |
| 8.4. methyl acetate | 4.5E-03 | 8.3E-03 | 2.2E-03 |
| 8.5. propyl acetate | 7.2E-02 | 9.7E-02 | 3.5E-02 |

NE; No effect

###

### Supplementary Table S3. Data for all variables used in the 6 prediction models.

| Compound | Compound name | In vivo category* | Neg.log (BMC20(M)) ^1,2^ | Neg.log (Conc at Emax  (M)) ^1,2^ | pH_conc at Emax ^1,2,3^ | Emax (% of CAP) ^1,2^ | Neg.log (BMC20+CZ(M)) ^1,3^ | Neg.log (Conc at Emax+CZ(M)) ^1,3^ | Emax+CZ (% of CAP) ^1,3^ | PC1 scores no CZ ^2^ | PC2 scores no CZ ^2^ | PC1 scores +CZ ^3^ | PC2 scores +CZ ^3^ | PC1 scores +/-CZ ^1^ | PC1 scores +/-CZ ^1^ |
| --- | --- | --- | --- | --- | --- | --- | --- | --- | --- | --- | --- | --- | --- | --- | --- |
| 7.1 | ammonium dodecyl sulfate | Irritating | 7.381 | 4.737 | 6.88 | 133 | 7.301 | 2.828 | 107 | -3.4303 | 0.6641 | 2.7617 | 0.6464 | -4.4324 | 0.4935 |
| 3.2 | butyric acid | Irritating | 7.175 | 5.686 | 7.05 | 95 | 7.064 | 5.209 | 75 | -3.0640 | 1.3341 | 3.0308 | 1.5067 | -4.3899 | 2.1064 |
| 5.2 | cyclohexylamine | Irritating | 6.692 | 4.83 | 7.11 | 132 | 6.393 | 4.352 | 106 | -3.2386 | 0.5417 | 3.0390 | 0.8517 | -4.4967 | 0.6401 |
| 5.3 | diethylamine | Irritating | 6.356 | 4.786 | 7.1 | 125 | 6.265 | 5.74 | 95 | -2.9782 | 0.6354 | 3.3292 | 1.3108 | -4.5416 | 1.1330 |
| 6.1 | phenol | Irritating | 4.575 | 1.881 | 6.92 | 114 | 2.975 | 1.881 | 100 | -1.1520 | 0.1745 | 0.9147 | 0.2042 | -1.4909 | -0.2159 |
| 7.2 | sodium dodecyl sulfate | Irritating | 4.163 | 2.368 | 7.39 | 123 | 3.659 | 1.891 | 120 | -1.3668 | -0.1081 | 1.5209 | -0.2275 | -2.0385 | -0.7498 |
| 2.3 | 2-hexanone | Moderate | 4.016 | 1.522 | 7.01 | 59 | 1.522 | 1.522 | 20 | 0.0943 | 0.8400 | -1.0744 | 0.9616 | 0.7704 | 1.6449 |
| 5.4 | ethylamine | Irritating | 3.948 | 1.653 | 9.26 | 109 | 3.697 | 1.653 | 109 | -0.8762 | -0.9680 | 1.4025 | -1.0819 | -1.5098 | -1.2530 |
| 5.5 | triethylamine | Irritating | 3.640 | 0.144 | 11.51 | 117 | 3.541 | 0.621 | 116 | -0.4648 | -2.5153 | 1.2646 | -2.5444 | -0.9898 | -2.8328 |
| 1.1 | 1-propanol | Non-irritant | 3.304 | 0.827 | 6.97 | 95 | 2.064 | 0.827 | 66 | -0.0207 | 0.1508 | -0.3589 | 0.2853 | 0.2032 | 0.0831 |
| 3.1 | acetic acid | Irritating | 3.186 | 2.621 | 4.29 | 65 | 3.164 | 2.142 | 56 | -0.0160 | 2.2576 | 0.0880 | 2.1357 | -0.2690 | 2.3041 |
| 5.1 | allylamine | Irritating | 3.182 | 0.829 | 11.5 | 134 | 3.027 | 1.306 | 116 | -0.8473 | -2.6404 | 1.3619 | -2.4134 | -1.3417 | -2.8772 |
| 5.6 | ammonia | Irritating | 2.980 | 0.554 | 11.52 | 118 | 2.700 | 1.031 | 88 | -0.4031 | -2.4760 | 0.6587 | -2.1609 | -0.5418 | -2.3106 |
| 2.2 | 2-heptanone | Moderate | 2.936 | 1.575 | 7.29 | 66 | 1.864 | 0.62 | 51 | 0.2992 | 0.5527 | -0.7461 | 0.2539 | 0.7246 | 0.7349 |
| 2.5 | 5-methyl-2-hexanone | Moderate | 2.926 | 0.625 | 7.4 | 66 | 1.525 | 0.625 | 59 | 0.6429 | 0.3006 | -0.7034 | 0.0800 | 0.9494 | 0.3588 |
| 2.6 | 5-methyl-3-heptanone | Moderate | 2.730 | 0.671 | 7.29 | 59 | 1.490 | 0.671 | 47 | 0.8252 | 0.4660 | -0.9177 | 0.2921 | 1.2161 | 0.7099 |
| 4.2 | formaldehyde | Irritating | 2.624 | 0.394 | 11.93 | 92 | 2.224 | 0.394 | 57 | 0.1948 | -2.3447 | -0.2418 | -2.1667 | 0.5347 | -1.7539 |
| 2.8 | cyclohexanone | Moderate | 2.386 | 0.492 | 7.13 | 112 | 2.122 | 0.969 | 81 | 0.0978 | -0.3064 | -0.0074 | 0.0555 | 0.0447 | -0.5989 |
| 8.4 | methyl acetate | Moderate | 2.346 | -0.1 | 6.79 | 108 | 1.702 | -0.322 | 94 | 0.4147 | -0.2023 | -0.4268 | -0.2669 | 0.5763 | -0.9708 |
| 2.7 | acetone | Non-irritant | 2.188 | 0.82 | 7.09 | 61 | 1.565 | 0.82 | 34 | 0.9157 | 0.5279 | -1.0760 | 0.5880 | 1.3653 | 0.9657 |
| 8.2 | allyl chloride | Moderate | 2.122 | 1.819 | 7.25 | 38 | 0.675 | -0.088 | 32 | 0.9631 | 0.9989 | -1.7190 | 0.2573 | 1.8908 | 1.3159 |
| 8.1 | allyl acetate | Irritating | 1.942 | 0.671 | 6.97 | 52 | 1.091 | 0.671 | 43 | 1.2049 | 0.6714 | -1.1303 | 0.4682 | 1.6168 | 0.9078 |
| 2.4 | 4-methyl-2-pentanone | Moderate | 1.836 | 0.574 | 7.39 | 84 | 1.106 | 0.574 | 64 | 0.7271 | -0.0197 | -0.7624 | -0.0204 | 1.0455 | -0.0982 |
| 2.1 | 2-butanone | Non-irritant | 1.388 | -0.047 | 7.32 | 84 | 0.792 | -0.047 | 58 | 1.0907 | -0.1502 | -1.2132 | -0.0744 | 1.6178 | -0.2587 |
| 1.2 | allyl alcohol | Irritating | 1.165 | 0.492 | 6.58 | 63 | 0.808 | 0.492 | 47 | 1.3583 | 0.6213 | -1.2577 | 0.5594 | 1.7924 | 0.6973 |
| 8.5 | propyl acetate | Moderate | 1.141 | 0.537 | 7.43 | 65 | 0.925 | 0.537 | 62 | 1.2864 | 0.1930 | -0.8740 | -0.0305 | 1.5267 | 0.1412 |
| 1.4 | isopropanol | Non-irritant | 0.863 | -0.115 | 7.1 | 53 | 0.582 | -0.115 | 44 | 1.8271 | 0.3747 | -1.5609 | 0.1725 | 2.3746 | 0.4418 |
| 4.1 | acetaldehyde | Moderate | 0.858 | 0.226 | 5.89 | 110 | 0.691 | 0.226 | 98 | 0.7894 | 0.1914 | -0.5361 | 0.1919 | 0.8671 | -0.7212 |
| 1.3 | ethanol | Non-irritant | 0.658 | -0.233 | 7.15 | 62 | 0.258 | -0.057 | 44 | 1.7887 | 0.1869 | -1.6393 | 0.1412 | 2.4019 | 0.2539 |
| 8.3 | dimethyl sulfoxide | Non-irritant | 0.520 | -0.148 | 7.11 | 92 | 0.482 | -0.148 | 63 | 1.3001 | -0.2226 | -1.2634 | -0.0861 | 1.7909 | -0.4965 |
| 1.5 | methanol | Non-irritant | 0.250 | -0.392 | 6.95 | 59 | -0.119 | -0.392 | 46 | 2.0376 | 0.2708 | -1.8633 | 0.1104 | 2.7328 | 0.2051 |

*) Irritating, RD50<700 ppm; moderate, RD50>700 but <10000 ppm; Non-irritant, RD50>10000 ppm

1) All in vitro variables (no CZ and +CZ), PC1 and PC2 scores (no CZ and +CZ) from Figure 11A.

2) In vitro variables (no CZ), PC1 and PC2 scores (no CZ) from Figure 11C.

3) In vitro variables (+CZ), PC1 and PC2 scores (+CZ) from Figure 11E.

### Supplementary Table S4. Probability for classification into the 2-class model by using all in vitro variables (no CZ and +CZ) in the random forest prediction

| **Chemical** | **In vivo RD_50_ (ppm)** | **In vivo class** | **fraction votes_class_01** | **fraction votes_class_2** | **# votes** | **predicted class** |
| --- | --- | --- | --- | --- | --- | --- |
| allyl acetate | 3 | 2 | 0.980 | 0.020 | 51 | 01 (UP) |
| allyl alcohol | 3 | 2 | 0.980 | 0.020 | 50 | 01 (UP) |
| formaldehyde | 5 | 2 | 1.000 | 0.000 | 54 | 01 (UP) |
| sodium dodecyl sulfate | 8 | 2 | 0.000 | 1.000 | 59 | 2 |
| allylamine | 9 | 2 | 0.000 | 1.000 | 44 | 2 |
| ammonium dodecyl sulfate | 10 | 2 | 0.000 | 1.000 | 45 | 2 |
| cyclohexylamine | 39 | 2 | 0.000 | 1.000 | 53 | 2 |
| ethylamine | 151 | 2 | 0.000 | 1.000 | 63 | 2 |
| phenol | 166 | 2 | 0.000 | 1.000 | 40 | 2 |
| triethylamine | 171 | 2 | 0.000 | 1.000 | 44 | 2 |
| diethylamine | 193 | 2 | 0.000 | 1.000 | 49 | 2 |
| butyric acid | 285 | 2 | 0.000 | 1.000 | 54 | 2 |
| acetic acid | 322 | 2 | 0.127 | 0.873 | 55 | 2 |
| ammonia | 546 | 2 | 0.051 | 0.949 | 39 | 2 |
| cyclohexanone | 757 | 01 | 0.585 | 0.415 | 53 | 01 |
| 5-methyl-3-heptanone | 759 | 01 | 1.000 | 0.000 | 43 | 01 |
| propyl acetate | 793 | 01 | 1.000 | 0.000 | 45 | 01 |
| methyl acetate | 829 | 01 | 0.963 | 0.037 | 54 | 01 |
| 2-heptanone | 893 | 01 | 1.000 | 0.000 | 53 | 01 |
| 5-methyl-2-hexanone | 1232 | 01 | 1.000 | 0.000 | 47 | 01 |
| allyl chloride | 1740 | 01 | 0.945 | 0.055 | 55 | 01 |
| 2-hexanone | 2555 | 01 | 0.434 | 0.566 | 53 | 2 (OP) |
| 4-methyl-2-pentanone | 3195 | 01 | 1.000 | 0.000 | 55 | 01 |
| acetaldehyde | 3574 | 01 | 1.000 | 0.000 | 42 | 01 |
| 1-propanol | 10381 | 01 | 0.690 | 0.310 | 58 | 01 |
| isopropanol | 11347 | 01 | 1.000 | 0.000 | 54 | 01 |
| 2-butanone | 17057 | 01 | 1.000 | 0.000 | 59 | 01 |
| ethanol | 20474 | 01 | 1.000 | 0.000 | 56 | 01 |
| methanol | 33368 | 01 | 1.000 | 0.000 | 58 | 01 |
| acetone | 50498 | 01 | 0.889 | 0.111 | 54 | 01 |
| dimethyl sulfoxide | N.A. | 01 | 1.000 | 0.000 | 61 | 01 |

UP, underpredicted

OP, overpredicted

N.A.; not available

### Supplementary Table S5. Probability for classification into the 2-class model by using in vitro variables for no CZ in the random forest prediction

| **Chemical** | **In vivo RD50 (ppm)** | **In vivo class** | **fraction votes_class_01** | **fraction votes_class_2** | **# votes** | **predicted class** |
| --- | --- | --- | --- | --- | --- | --- |
| allyl acetate | 3 | 2 | 1.000 | 0.000 | 51 | 01 (UP) |
| allyl alcohol | 3 | 2 | 0.980 | 0.020 | 50 | 01 (UP) |
| formaldehyde | 5 | 2 | 0.981 | 0.019 | 54 | 01 (UP) |
| sodium dodecyl sulfate | 8 | 2 | 0.000 | 1.000 | 59 | 2 |
| allylamine | 9 | 2 | 0.023 | 0.977 | 44 | 2 |
| ammonium dodecyl sulfate | 10 | 2 | 0.000 | 1.000 | 45 | 2 |
| cyclohexylamine | 39 | 2 | 0.000 | 1.000 | 53 | 2 |
| ethylamine | 151 | 2 | 0.079 | 0.921 | 63 | 2 |
| phenol | 166 | 2 | 0.075 | 0.925 | 40 | 2 |
| triethylamine | 171 | 2 | 0.045 | 0.955 | 44 | 2 |
| diethylamine | 193 | 2 | 0.000 | 1.000 | 49 | 2 |
| butyric acid | 285 | 2 | 0.019 | 0.981 | 54 | 2 |
| acetic acid | 322 | 2 | 0.527 | 0.473 | 55 | 01 (UP) |
| ammonia | 546 | 2 | 0.154 | 0.846 | 39 | 2 |
| cyclohexanone | 757 | 01 | 0.830 | 0.170 | 53 | 01 |
| 5-methyl-3-heptanone | 759 | 01 | 0.977 | 0.023 | 43 | 01 |
| propyl acetate | 793 | 01 | 1.000 | 0.000 | 45 | 01 |
| methyl acetate | 829 | 01 | 0.944 | 0.056 | 54 | 01 |
| 2-heptanone | 893 | 01 | 0.868 | 0.132 | 53 | 01 |
| 5-methyl-2-hexanone | 1232 | 01 | 1.000 | 0.000 | 47 | 01 |
| allyl chloride | 1740 | 01 | 0.764 | 0.236 | 55 | 01 |
| 2-hexanone | 2555 | 01 | 0.226 | 0.774 | 53 | 2 (OP) |
| 4-methyl-2-pentanone | 3195 | 01 | 1.000 | 0.000 | 55 | 01 |
| acetaldehyde | 3574 | 01 | 0.929 | 0.071 | 42 | 01 |
| 1-propanol | 10381 | 01 | 0.276 | 0.724 | 58 | 2 (OP) |
| isopropanol | 11347 | 01 | 1.000 | 0.000 | 54 | 01 |
| 2-butanone | 17057 | 01 | 1.000 | 0.000 | 59 | 01 |
| ethanol | 20474 | 01 | 1.000 | 0.000 | 56 | 01 |
| methanol | 33368 | 01 | 1.000 | 0.000 | 58 | 01 |
| acetone | 50498 | 01 | 0.981 | 0.019 | 54 | 01 |
| dimethyl sulfoxide | N.A. | 01 | 1.000 | 0.000 | 61 | 01 |

UP, underpredicted

OP, overpredicted

N.A.; not available

### Supplementary Table S6. Probability for classification into the 3-class model by using all in vitro variables (no CZ and +CZ) in the random forest prediction.

| **Chemical** | **In vivo RD50 (ppm)** | **In vivo class** | **fraction votes_class_0** | **fraction votes_class_1** | **fraction votes_class_2** | #votes | **predicted class** |
| --- | --- | --- | --- | --- | --- | --- | --- |
| allyl acetate | 3 | 2 | 0.412 | 0.490 | 0.098 | 51 | 1 (UP) |
| allyl alcohol | 3 | 2 | 0.540 | 0.300 | 0.160 | 50 | 0 (UPx2) |
| formaldehyde | 5 | 2 | 0.019 | 0.981 | 0.000 | 54 | 1 (UP) |
| sodium dodecyl sulfate | 8 | 2 | 0.000 | 0.000 | 1.000 | 59 | 2 |
| allylamine | 9 | 2 | 0.000 | 0.000 | 1.000 | 44 | 2 |
| ammonium dodecyl sulfate | 10 | 2 | 0.000 | 0.000 | 1.000 | 45 | 2 |
| cyclohexylamine | 39 | 2 | 0.000 | 0.000 | 1.000 | 53 | 2 |
| ethylamine | 151 | 2 | 0.000 | 0.000 | 1.000 | 63 | 2 |
| phenol | 166 | 2 | 0.000 | 0.000 | 1.000 | 40 | 2 |
| triethylamine | 171 | 2 | 0.000 | 0.000 | 1.000 | 44 | 2 |
| diethylamine | 193 | 2 | 0.000 | 0.000 | 1.000 | 49 | 2 |
| butyric acid | 285 | 2 | 0.000 | 0.000 | 1.000 | 54 | 2 |
| acetic acid | 322 | 2 | 0.018 | 0.091 | 0.891 | 55 | 2 |
| ammonia | 546 | 2 | 0.000 | 0.051 | 0.949 | 39 | 2 |
| cyclohexanone | 757 | 1 | 0.094 | 0.302 | 0.604 | 53 | 2 (OP) |
| 5-methyl-3-heptanone | 759 | 1 | 0.070 | 0.907 | 0.023 | 43 | 1 |
| propyl acetate | 793 | 1 | 0.178 | 0.822 | 0.000 | 45 | 1 |
| methyl acetate | 829 | 1 | 0.204 | 0.648 | 0.148 | 54 | 1 |
| 2-heptanone | 893 | 1 | 0.000 | 1.000 | 0.000 | 53 | 1 |
| 5-methyl-2-hexanone | 1232 | 1 | 0.000 | 1.000 | 0.000 | 47 | 1 |
| allyl chloride | 1740 | 1 | 0.655 | 0.164 | 0.182 | 55 | 0 (UP) |
| 2-hexanone | 2555 | 1 | 0.208 | 0.189 | 0.604 | 53 | 2 (OP) |
| 4-methyl-2-pentanone | 3195 | 1 | 0.000 | 1.000 | 0.000 | 55 | 1 |
| acetaldehyde | 3574 | 1 | 0.214 | 0.738 | 0.048 | 42 | 1 |
| 1-propanol | 10381 | 0 | 0.000 | 0.621 | 0.379 | 58 | 1 (OP) |
| isopropanol | 11347 | 0 | 0.963 | 0.037 | 0.000 | 54 | 0 |
| 2-butanone | 17057 | 0 | 0.169 | 0.831 | 0.000 | 59 | 1 (OP) |
| ethanol | 20474 | 0 | 1.000 | 0.000 | 0.000 | 56 | 0 |
| methanol | 33368 | 0 | 1.000 | 0.000 | 0.000 | 58 | 0 |
| acetone | 50498 | 0 | 0.000 | 0.852 | 0.148 | 54 | 1 (OP) |
| dimethyl sulfoxide | N.A. | 0 | 0.754 | 0.246 | 0.000 | 61 | 0 |

UP, underpredicted by 1 class

UPx2, underpredicted by 2 classes

OP, overpredicted by 1 class

N.A.; not available

### Supplementary Table S7. Probability for classification into the 3-class model by using in vitro variables for +CZ in the random forest prediction.

| **Chemical** | **In vivo RD50 (ppm)** | **In vivo class** | **fraction votes_class_0** | **fraction votes_class_1** | **fraction votes_class_2** | #votes | **predicted class** |
| --- | --- | --- | --- | --- | --- | --- | --- |
| allyl acetate | 3 | 2 | 0.431 | 0.392 | 0.176 | 51 | 0 (UPx2) |
| allyl alcohol | 3 | 2 | 0.440 | 0.280 | 0.280 | 50 | 0 (UPx2) |
| formaldehyde | 5 | 2 | 0.037 | 0.944 | 0.019 | 54 | 1 (UP) |
| sodium dodecyl sulfate | 8 | 2 | 0.000 | 0.000 | 1.000 | 59 | 2 |
| allylamine | 9 | 2 | 0.000 | 0.000 | 1.000 | 44 | 2 |
| ammonium dodecyl sulfate | 10 | 2 | 0.000 | 0.000 | 1.000 | 45 | 2 |
| cyclohexylamine | 39 | 2 | 0.000 | 0.000 | 1.000 | 53 | 2 |
| ethylamine | 151 | 2 | 0.000 | 0.000 | 1.000 | 63 | 2 |
| phenol | 166 | 2 | 0.000 | 0.000 | 1.000 | 40 | 2 |
| triethylamine | 171 | 2 | 0.000 | 0.000 | 1.000 | 44 | 2 |
| diethylamine | 193 | 2 | 0.000 | 0.000 | 1.000 | 49 | 2 |
| butyric acid | 285 | 2 | 0.000 | 0.000 | 1.000 | 54 | 2 |
| acetic acid | 322 | 2 | 0.000 | 0.018 | 0.982 | 55 | 2 |
| ammonia | 546 | 2 | 0.000 | 0.026 | 0.974 | 39 | 2 |
| cyclohexanone | 757 | 1 | 0.170 | 0.189 | 0.642 | 53 | 2 (OP) |
| 5-methyl-3-heptanone | 759 | 1 | 0.023 | 0.953 | 0.023 | 43 | 1 |
| propyl acetate | 793 | 1 | 0.044 | 0.956 | 0.000 | 45 | 1 |
| methyl acetate | 829 | 1 | 0.241 | 0.630 | 0.130 | 54 | 1 |
| 2-heptanone | 893 | 1 | 0.000 | 1.000 | 0.000 | 53 | 1 |
| 5-methyl-2-hexanone | 1232 | 1 | 0.000 | 1.000 | 0.000 | 47 | 1 |
| allyl chloride | 1740 | 1 | 0.909 | 0.091 | 0.000 | 55 | 0 (UP) |
| 2-hexanone | 2555 | 1 | 0.321 | 0.132 | 0.547 | 53 | 2 (OP) |
| 4-methyl-2-pentanone | 3195 | 1 | 0.036 | 0.964 | 0.000 | 55 | 1 |
| acetaldehyde | 3574 | 1 | 0.262 | 0.476 | 0.262 | 42 | 1 |
| 1-propanol | 10381 | 0 | 0.000 | 0.828 | 0.172 | 58 | 1 (OP) |
| isopropanol | 11347 | 0 | 0.778 | 0.204 | 0.019 | 54 | 0 |
| 2-butanone | 17057 | 0 | 0.153 | 0.847 | 0.000 | 59 | 1 (OP) |
| ethanol | 20474 | 0 | 0.804 | 0.196 | 0.000 | 56 | 0 |
| methanol | 33368 | 0 | 0.948 | 0.052 | 0.000 | 58 | 0 |
| acetone | 50498 | 0 | 0.019 | 0.685 | 0.296 | 54 | 1 (OP) |
| dimethyl sulfoxide | N.A. | 0 | 0.820 | 0.180 | 0.000 | 61 | 0 |

UP, underpredicted by 1 class

UPx2, underpredicted by 2 classes

OP, overpredicted by 1 class

N.A.; not available
